# Effectiveness and safety of single anti-seizure medication as adjunctive therapy for drug-resistant focal epilepsy based on network meta-analysis

**DOI:** 10.3389/fphar.2025.1500475

**Published:** 2025-04-25

**Authors:** Nian-Jia Deng, Xin-Yi Li, Zhi-Xin Zhang, Chen-Yang Xian-Yu, Yu-Ting Tao, Yu-Tong Ma, Hui-Jun Li, Teng-Yu Gao, Xin Liu, Jie Luo, Chao Zhang, Sheng-Li Hu

**Affiliations:** ^1^ Center for Evidence-Based Medicine and Clinical Research, Hubei Provincial Clinical Research Center of Central Nervous System Repair and Functional Reconstruction, Taihe Hospital, Hubei University of Medicine, Shiyan, Hubei, China; ^2^ Department of Neurosurgery, Hubei Provincial Clinical Research Center of Central Nervous System Repair and Functional Reconstruction, Taihe Hospital, Hubei University of Medicine, Shiyan, Hubei, China

**Keywords:** drug-resistant focal seizures, anti-seizure medication, topiramate, levetiracetam, gabapentin, pregabalin

## Abstract

**Objective:**

To evaluated the effectiveness and safety of single anti-seizure medication (ASM) when used as adjunctive therapy for drug-resistant focal epilepsy.

**Methods:**

We conducted a comprehensive search of PubMed, EMbase, and the Cochrane Library from their inception until 12 February, 2025, to identify randomized controlled trials (RCTs) meeting our criteria. The trials were analyzed for their use of ASMs in treating drug-resistant focal epilepsy. Inclusion criteria comprised: 1) Participants aged 12 years or older with drug-resistant focal epilepsy; 2) Incorporation of an additional single ASM as an adjunct to the existing antiepileptic treatment regimen; 3) Comparison with placebo or continuation of the original antiepileptic regimen without a new ASM; 4) Primary outcome as a 50% response rate, with safety as a secondary outcome, encompassing dizziness, somnolence, headache, ataxia, diplopia, fatigue, and nausea; and 5) Study design limited to RCTs. The surface under the cumulative ranking curve (SUCRA) was employed to rank the effectiveness and safety of the ASMs.

**Results:**

A total of 53 RCTs involving 17 ASMs as adjunctive therapy and placebo were analyzed. Compared to placebo, the following ASMs demonstrated statistically significant effectiveness in achieving a 50% response rate: brivaracetam (RR = 2.07, 95% CI: 1.53–2.81), cenobamate (RR = 2.12, 95% CI: 1.56–2.88), eslicarbazepine acetate (RR = 1.95, 95% CI: 1.41–2.70), gabapentin (RR = 2.30, 95% CI: 1.76–3.02), lacosamide (RR = 2.22, 95% CI: 1.47–3.35), lamotrigine (RR = 1.55, 95% CI: 1.00–2.40), levetiracetam (RR = 2.43, 95% CI: 1.88–3.15), oxcarbazepine (RR = 3.03, 95% CI: 2.08–4.40), perampanel (RR = 1.72, 95% CI: 1.21–2.44), pregabalin (RR = 2.06, 95% CI: 1.70–2.50), rufinamide (RR = 2.28, 95% CI: 1.20–4.31), tiagabine (RR = 4.07, 95% CI: 2.03–8.18), topiramate (RR = 3.10, 95% CI: 2.44–3.95), vigabatrin (RR = 2.34, 95% CI: 1.58–3.46), and zonisamide (RR = 2.40, 95% CI: 1.76–3.27). Based on SUCRA rankings, tiagabine (92.7%) exhibited the most favorable therapeutic outcome, followed by topiramate (87.3%), oxcarbazepine (83%), and levetiracetam (62.8%). The ASMs with the least favorable therapeutic effects were placebo (1.1%), lamotrigine (17.8%), and perampanel (24.7%).

**Conclusion:**

The network meta-analysis revealed topiramate, tiagabine, oxcarbazepine, and levetiracetam as the four most effective adjuvant ASM treatments for drug-resistant focal epilepsy. However, it is noteworthy that topiramate and oxcarbazepine were associated with a higher incidence of somnolence. Additionally, comprehensive safety data for tiagabine and levetiracetam are lacking, necessitating further research. Larger studies are required to solidify these findings and better understand the safety profiles of all involved ASMs.

## Introduction

Epilepsy was stands as one of the most prevalent brain disorders worldwide, impacting over 70 million individuals across all age groups, from infants and young children to the elderly, to varying degrees. The most frequent form of epilepsy in humans was focal epilepsy, which comprises more than half of all cases and poses the greatest therapeutic challenge when treated with anti-epileptic medications (Gooley et al., 2022; Engel, 2004). Focal seizures typically originated in a confined area of the cerebral cortex and subsequently propagate to adjacent regions, encompassing both the surrounding cortical tissue and subcutaneous structures (Jenssen et al., 2011). The most typical pathological conditions associated with focal epilepsy include traumatic brain injuries, tumors, and vascular malformations (Bernasconi and Bernasconi, 2022). Meanwhile, drug-resistant epilepsy referred to cases where seizures persist despite adjustments to anti-seizure medication (ASM) therapy, rendering seizure freedom highly improbable with further pharmacological interventions.

Over the past few decades, remarkable progress had been achieved in the treatment of epilepsy, with approximately 30 ASMs now clinically available. These ASMs had played a pivotal role in decreasing the frequency and severity of seizures, ultimately enhancing the quality of life for epilepsy patients (Löscher and Klein, 2021). A study revealed that topiramate, levetiracetam, pregabalin, and oxcarbazepine offered advantages over other ASMs in terms of adverse reactions and treatment risks. Conversely, rufinamide demonstrated suboptimal treatment effectiveness and a high risk of severe, urgent headaches (Zhao et al., 2017). Another meta-analysis (Hu et al., 2018) found that brivaracetam, levetiracetam, oxcarbazepine, vigabatrin, and topiramate exhibited reliable effectiveness, with levetiracetam being the most well-tolerated. Additionally, the study suggested that levetiracetam, vigabatrin, and gabapentin offered the best balance of short-term effectiveness and tolerability, while oxcarbazepine was effective but poorly tolerated (Bodalia et al., 2013). Despite consistent findings highlighted levetiracetam’s effectiveness, the efficacy of other ASMs as adjunctive therapy remained controversial due to factors such as limited sample sizes, unclear outcome definitions, and variations in patient populations. To provided clinicians with more authoritative and efficient guidelines, an updated and comprehensive network meta-analysis was conducted to evaluate the effectiveness and safety of adding a new single ASM to an existing anti-epileptic regimen for drug-resistant focal epilepsy among the various available options.

## Methods

This study was conducted in accordance with the extended Preferred Reporting Items for Systematic Reviews and Meta-analyses (PRISMA) guidelines specifically tailored for network meta-analyses of healthcare interventions (Hutton et al., 2015).

### Search strategy

As of 12 February, 2025, we involved a network meta-analysis by searching to identify related RCTs in the PubMed, EMbase and Cochrane Library. The MeSH and keywords used in the search were “drug-resistant,” “medication-resistant,” “intractable,” “refractory,” “uncontrolled,” “drug refractory,” “pharmacoresistant,” “complex,” “partial,” “partial-onset,” “focal,” “epilepsy,” “seizure,” and “randomized controlled trial.” The literature search strategies were showed in Supplementary Method S1.

### Inclusion and exclusion criteria

The inclusion criteria were as follows: 1) Population: Participants with drug-resistant focal epilepsy (age ≥12 years). 2) Intervention: Incorporating an additional single ASM as an adjunct to the existing antiepileptic treatment regimen. 3) Comparison: Placebo or no new ASM as adjunctive therapy to an existing anti-epileptic regimen. 4) Outcomes: All studies included at least one effectiveness or safety outcomes. Effectiveness outcome was defined as 50% response rate, and was used as the primary outcome. Safety outcomes were used as the secondary outcomes, including dizziness, somnolence, headache, ataxia, diplopia, fatigue and nausea. 5) Study designs: Randomised controlled trials (RCTs).

The exclusion criteria included duplicate studies, no specific descriptions of ASMs as adjunctive therapy, studies with missing data, conference proceedings, and publications that are solely accessible in the abstract form.

### Data collection and processing

Five authors (Nian-Jia Deng, Xin-Yi Li, Zhi-Xin Zhang, Chen-Yang Xian-Yu, Yu-Ting Tao), in consensus, independently filtrate the literature and strictly extracted data in accordance with the predetermined inclusion criteria. Any potential conflicts or differences of opinion among the authors were resolved through a process of deliberation and consultation involving a fourth author (Yu-Tong Ma). The fundamental information of each study was extracted, including the year, sex ratio of participants, mean age, median duration of epilepsy (years), main inclusion criteria, comparison measures, and sample size.

### Quality assessment

Two reviewers independently assessed the risk of bias of the included studies (RoB-2) (Sterne et al., 2019). The RoB-2 evaluated studies in five domains: bias arising from the randomization process, bias due to deviations from intended interventions, bias due to missing outcome data, bias in outcome measurements, and bias in the selection of the reported results. There were “yes,” “probably yes,” “probably no,” “no,” and “no information” to answer the signal questions in the above domains. Notably, the consequences for bias risk were the same for “yes” and “probably yes” replies as they were for “no” and “probably no”. Additionally, the “probably” versions would typically imply that a judgment had been made. Following the completion of the signaling questions, a risk-of-bias assessment was made, and each domain was given one of three levels: low risk of bias, some concerns or high risk of bias.

### Statistical analysis

All dichotomous outcomes were employed for relative risk (RR) with 95% confidence intervals (CI), with a significant level of P < 0.05. I^2^ was used to detect the magnitude of heterogeneity. Additionally, the I^2^ statistic was used, where I^2^ values of ≥40% were indicative of significant heterogeneity (Higgins and James, 2011), the random effects model was employed. Otherwise, the fixed effects model was used. Network meta-analyses offer trustworthy proof for both direct and indirect comparisons of many interventions (Lu and Ades, 2004). The “loop inconsistency” method was employed for test of consistency equations when the treatment effects around a loop (Song et al., 2011). By definition, the surface under the cumulative ranking curve (SUCRA) values reflect the effectiveness and safety of ASMs as adjunctive therapy; thus, a rank plot with larger SUCRA scores implies more effective or safe ASMs as adjunctive therapy (Rücker and Schwarzer, 2015). Furthermore, a network funnel plot was used to detect any potential publication bias. All statistical analyses were conducted using STATA 15.0 and R 4.2.2, and it obtained a copyright license.

## Results

### Search results

**FIGURE 1:**
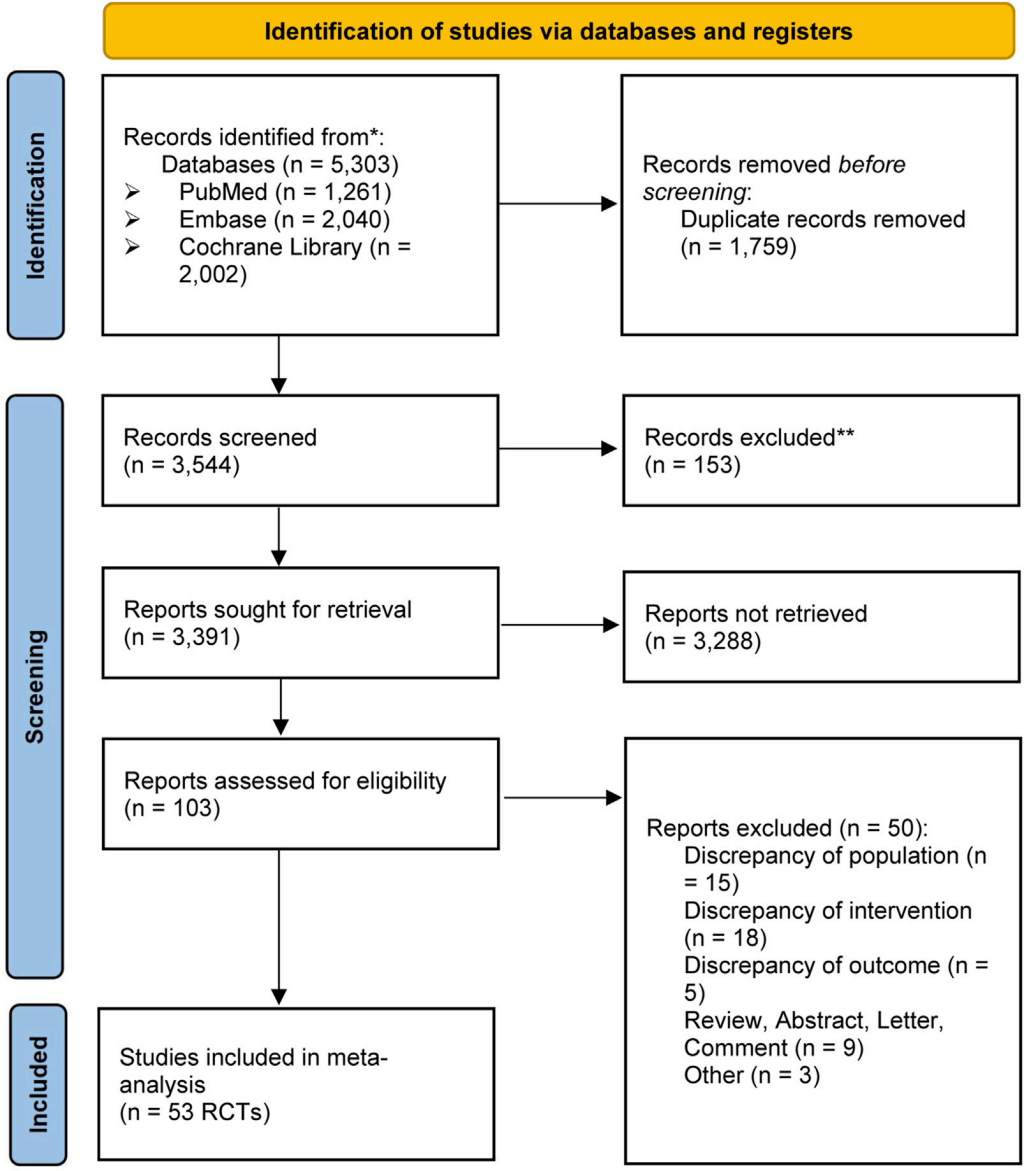
Study selection.

In total, 5,303 relevant studies were retrieved, of which 1,759 were removed as duplicates. For participants who met the diagnostic standard for drug-resistant focal epilepsy, quantitative data was obtained for the network meta-analysis by scrutinizing the relevant literature titles, abstracts and full-text evaluations. Finally, a total of 53 studies comprising 13,700 participants with 17 ASMs as adjunctive therapy and placebo were involved in this study (Figure 1).

### Basic characteristics and quality assessment

Table 1 showed the primary attributes characteristics of the included studies, incorporating the quantity of study (n = 53), study year, sex ratio of participants, mean age, median duration of epilepsy (years), main inclusion criteria, comparison measures, and sample size. Active ASMs as adjunctive therapy, including brivaracetam, cenobamate, eslicarbazepine acetate, gabapentin, lacosamide, lamotrigine, levetiracetam, natalizumab, oxcarbazepine, perampanel, pregabalin, remacemid, rufinamide, tiagabine, topiramate, vigabatrin and zonisamide were incorporated in the network meta-analysis. An assessment of the risk of bias from randomized trials was conducted utilizing the latest RoB-2 assessment tool (Supplementary Table S1).

**TABLE 1:** Basic information of included studies.

Study	Year	PMID	Main inclusion criteria	Comparisons	Sample	Male (%)	Age, mean (range)	Duration of epilepsy (year)
Anhut	1994	8082624	≥12 years, 40–110 kg were eligible, as were women of childbearing potential using an adequate form of contraception. Patients with partial seizures who failed to respond to standard ASM therapy at maximum tolerated dosages were eligible for this study. Specifically, patients had an average of four clearly recognizable partial seizures per month during the 3 months before screening, despite treatment with one or two currently available ASMs. The dosage of these ASMs was stable during the 3 months before screening	Placebo	109	56.2	12–67	>19
				Gabapentin 900 mg/day	111			21
				Gabapentin 1,200 mg/day	52			14
Arroyo	2004	14692903	≥18 years, 50–135 kg, with the International League Against Epilepsy–defined partial seizures were allowed to enter	Placebo	96	50.5	17–73	22.78 ± 13.58
				Pregabalin 150 mg/day	99			24.8 ± 12.65
				Pregabalin 600 mg/day	92			25.06 ± 11.63
Barcs	2000	11114219	Men and women aged 15–65 years were eligible. Patients had to experience an average of at least four partial seizures per month during the 8-week baseline phase preceding the double-blind treatment phase while maintained on one to three concomitant ASMs	Placebo	173	44.5	34.3 (15–65)	NA
				Oxcarbazepine 600 mg/day	168	51.2	34.6 (15–65)	NA
				Oxcarbazepine 1,200 mg/day	177	45.2	33.8 (16–64)	NA
				Oxcarbazepine 2,400 mg/day	174	56.3	35.2 (15–66)	NA
Baulac	2010	20696552	Men and nonpregnant, nonlactating women, ≥18 years of age, ≥40 kg, with a diagnosis of epilepsy with partial seizures were enrolled in the study. Diagnosis of epilepsy must have been consistent with results of an electroencephalogram performed within 2 years prior to randomization. The patients’ partial seizures had to be refractory to treatment	Placebo	140	55 (39.3)	39.1 (11.2)	23.4 (12.2)
				Pregabalin 300/600 mg/day	152	78 (51.3)	39.8 (11.2)	23.1 (13.5)
				Lamotrigine 300/400 mg/day	141	77 (54.6)	39.4 (11.4)	23.1 (13.6)
Ben-Menachem	1996	8641230	Men and women aged 18–65 years with a history of partial seizures which had not responded to treatment with one or two ASMs were selected for entry into the baseline phase of the study. Patients had to have at least eight partial seizures during the 8-week baseline period while maintained on therapeutic doses and plasma concentrations of one or two appropriate ASMs. During this phase, the longest allowable seizure-free period was 3 weeks, and only one such period was permitted	Placebo	28	84	37.2	NA
				Topiramate 800 mg/day	28			NA
Ben-Menachem	1997	9092955	Patients between 18 and 65 years of age who were experiencing four or more seizures per month while receiving one or two standard ASMs during an 8-week baseline period were eligible for randomization to add-on therapy with Topiramate or placebo	Placebo	24	NA	30	NA
				Topiramate 400 mg/day	23		31	
				Placebo	30		30	
				Topiramate 600 mg/day	30		31	
				Placebo	28		36	
				Topiramate 800 mg/day	28		40	
Ben-Menachem	2010	20299189	≥18 years, assessed as being in general good health; diagnosed with simple or complex partial-onset seizures (with or without secondary generalization) for a minimum of 12 months prior to screening	Placebo	100	49.1	18–69	25.4 ± 13.06
				Eslicarbazepine acetate 400 mg/day	96			24.7 ± 11.52
				Eslicarbazepine acetate 800 mg/day	101			22.4 ± 11.63
				Eslicarbazepine acetate 1,200 mg/day	98			23.0 ± 12.90
Beydoun	2005	15699378	≥18 years, 50–135 kg, with inadequately controlled partial-onset seizures diagnosed by patient history and a recent EEG (within the preceding 2 years). To be eligible, patients had to experience a minimum of six partial-onset seizures during a prospective 8-week baseline period, with no 28-day seizure-free period, while maintained on stable doses of one to three ASMs. Patients also had to have failed two or more ASMs at maximally tolerated doses	Placebo	98	50	17–82	23.5 ± 11.9
				Pregabalin 600 mg/day	215			26.8 ± 13.0
Biton	2011	20887365	Eligible patients were male or female, aged 12–80 years. Those who had ≧6 seizures during the 56 days, with no 21-day seizure free periods, were eligible for randomization into the double-blind phase of the study	Placebo	175	83	38.1	NA
				Rufinamide 3,200 mg/day	160	84	36.4	NA
Brodie	2004	15511696	18–59 years had a history of refractory partial seizures (at least four seizures per month in the previous 4 months), and were being treated with one or two ASMs, but not more than two of the following: phenytoin, carbamazepine, sodium valproate, phenobarbital, or primidone. In addition, patients had to be capable of counting the number of seizures that they experienced, because their record of seizure activity was an important component of the study data	Placebo	71	59	18–59	NA
				Zonisamide 400 mg/day	73			NA
Brodie	2005	15660766	≥12 years, with partial seizures with or without secondary generalization unsatisfactorily controlled despite a stable regimen of one to three ASMs. Seizures were classified according to International League Against Epilepsy (ILAE) criteria into simple partial (SP) seizures, complex partial (CP) seizures, and partial seizures with secondary generalization (SGS)	Placebo	120	57.6	12–77	20.4 (1.8–48.8)
				Zonisamide 100 mg/day	56			23.4 (0.42–56)
				Zonisamide 300 mg/day	55			15.7 (0.56–55.8)
				Zonisamide 500 mg/day	118			18.9 (0.92–64.7)
Bruni	2000	10777431	16–50 years, with a definite diagnosis of complex partial seizures or partial seizures with secondary generalization were entered. This diagnosis was confirmed by documented focal EEG abnormalities. Patients were required to have a minimum of six complex partial seizures or partial seizures secondarily generalized over the 8-week period preceding entry	Placebo	53	55	18–50	19 ± 1.4
				Vigabatrin 3,000 mg/day	58			21 ± 1.2
Cereghino	2000	10908898	16–70 years, experienced uncontrolled partial seizures with or without becoming secondarily generalized for at least 2 years. Patients had to have a minimum of 12 partial seizures within 12 weeks before study selection, with a minimum of two partial seizures occurring per 4 weeks during the baseline period. Patients must have received at least two marketed ASMs, either simultaneously or consecutively	Placebo	95	60.5	16–70	>2
				Levetiracetam 1,000 mg/day	98			
				Levetiracetam 3,000 mg/day	101			
Chadwick	2000	11162751	This was a two-center, double-blind, randomized, three-way parallel group comparison of adjunctive remacemide hydrochloride, and placebo, over 28 days, in patients with epilepsy	Placebo	14	85.7%	40.4 (23–66)	21.9 (4–54)
				Remacemide 300 mg/day	13	69.2%	36.3 (20–53)	23.5 (4–41)
				Remacemide 400 mg/day	13	92.3%	36.2 (22–60)	22.2 (4–40)
Chung	2020	32409485	Patients were adults 18–65 years of age with a diagnosis of treatment-resistant focal (partial-onset) epilepsy, as defined by the International League Against Epilepsy. All seizure diagnoses were confirmed by an independent review from The Epilepsy Study Consortium. Patients must have been taking 1 to 3 ASMs at stable doses for at least 12 weeks before randomization	Placebo	109	58 (53.2)	38 (18, 59)	21.1 (24.2, 60.75)
				Cenobamate 200 mg/day	113	55 (48.7)	36 (18, 61)	19.95 (2.33, 52.5)
Faught	1997	9092954	18–65 years patients were required to have experienced at least four seizures per month during a 3-month baseline period while receiving one or two standard ASMs at therapeutic levels	Placebo	45	80	34	NA
				Topiramate 200 mg/day	45			NA
				Topiramate 400 mg/day	42			NA
				Topiramate 600 mg/day	43			NA
Faught	1997			Placebo	47			NA
				Topiramate 600 mg/day	44			NA
				Topiramate 800 mg/day	44			NA
				Topiramate 1,000 mg/day	42			NA
Faught	1996	8649570	18–65 years patients were further required to have experienced at least 12 partial seizures during the 12-week baseline period preceding the double-blind study phase while maintained at therapeutic ASM plasma concentrations	Placebo	45	80	36.2 (19–68)	NA
				Topiramate 200 mg/day	45	64.4	38.6 (19–67)	NA
				Topiramate 400 mg/day	45	86.7	38.9 (19–61)	NA
				Topiramate 600 mg/day	46	84.8	33.8 (20–58)	NA
French	1996	8559421	18–60 years, with a diagnosis of complex partial seizures, with or without secondary generalization, whose seizures had been unsatisfactorily controlled with currently available anti-epilepsy medication, were eligible for participation in the study. Eligibility required that all patients studied had at least six documented complex partial seizures during the last 8 weeks of a 12-week pre-study screening period, despite a stable regimen of treatment of at least one, but not more than two, currently available anti-epileptic agents	Placebo	90	44	18–60	NA
				Vigabatrin 3,000 mg/day	92			NA
French	2003	12771254	12–70 years, had experienced at least three observable partial seizures in the month prior to screening and six partial seizures in the 8 weeks between screening and baseline; their disease was refractory to at least two ASMs at maximally tolerated doses; and they were currently receiving at least one but no more than three ASMs	Placebo	100	48.1	12–75	24 ± 10
				Pregabalin 50 mg/day	88			25 ± 11.8
				Pregabalin 150 mg/day	86			24 ± 12.8
				Pregabalin 300 mg/day	90			26.2 ± 13.5
				Pregabalin 600 mg/day	89			25.5 ± 13.7
French	2010	20592253	Patients were included if they were aged 16–65 years with well-characterized focal epilepsy/epileptic syndrome (International League Against Epilepsy classification, 1989) 11 experiencing at least 4 partial-onset seizures during a 4-week prospective baseline period and taking 1 or 2 concomitant ASMs maintained at stable dose from at least 1 month before screening and throughout the study	Placebo	54	24 (44.4)	33.6 (11.3)	21.7 (13.0)
				Brivaracetam 5 mg/day	50	30 (60.0)	32.7 (12.2)	16.0 (11.5)
				Brivaracetam 20 mg/day	52	28 (53.8)	35.3 (13.7)	22.9 (13.5)
				Brivaracetam 50 mg/day	52	28 (53.8)	30.9 (11.6)	19.1 (10.8)
French	2014	24962242	Patients were ≥18 years. A minimum of six partial seizures with an observable component with no 28-day period free of partial seizures during the 8-week baseline was required for randomization	Placebo	109	44.5	38.7 (18–72)	NA
				Pregabalin 165 mg/day	98	47	37.9 (18–70)	NA
				Pregabalin 330 mg/day	111	51.3	39.6 (18–75)	NA
French	2016	27521437	Key criteria were age 18–80 years, a diagnosis of epilepsy with partial-onset seizures (equivalent to the 2010 ILAE classification1of focal seizures) that had been inadequately controlled with 2 to 5 prior ASMs, and receiving 1 or 2 standard ASMs (other than pregabalin or gabapentin) with a minimum of 4 partial-onset seizures	Pregabalin 150, 300, 450, and 600 mg/day	241	127 (52.7)	34.9 (13.0)	19.8 (0.1–78.1)
				Gabapentin 300, 600, 1,200, 1,500, and 1,800 mg/day	241	130 (53.9)	35.3 (12.9)	19.9 (0.0–62.1)
French	2021	34521687	Participants aged 18–75 years were eligible for enrolment if they had a clinical diagnosis of focal epilepsy (confirmed by an independent epilepsy review committee) and met the International League Against Epilepsy’s 2010 definition of drug resistance. In addition, participants must have experienced ≥6 seizures during the baseline period, with no more than 21 consecutive seizure-free days, and been on a stable regimen of 1–5 ASDs during the 4 weeks before the screening visit and throughout the baseline period	Placebo	34	18 (53)	39.1 (12.17)	19.6 (14.69)
				Natalizumab 300 mg/day	32	18 (56)	42.8 (14.56)	19.7 (13.30)
Gil-Nagel	2009	19832771	≥18 years, assessed as being in general good health, other than epilepsy; diagnosed with simple or complex partial seizures (with or without secondary generalization) for a minimum of 12 months prior to screening; experienced at least four partial-onset seizures	Placebo	87	44.8	≧18	23.8 ± 13.03
				Eslicarbazepine acetate 800 mg/day	85			22.5 ± 11.78
				Eslicarbazepine acetate 1,200 mg/day	80			23.0 ± 13.01
Guberman	2002	12225311	18–65 years had at least three partial-onset seizures, with or without secondary generalization, within the 4-week baseline	Placebo	91	50	36 (18–67)	NA
				Topiramate 200 mg/day	168	46	37 (18–64)	NA
Hogan	2014	25461205	18–75 years with a confirmed diagnosis of partial-onset seizures (for ≥1 year) with a minimum of eight partial-onset seizures (with or without secondary generalization) and no more than 21 consecutive seizure-free days during the 8-week baseline phase	Placebo	63	52.8	37.6	NA
				Topiramate 200 mg/day	52	53.2	37.6	NA
Hong	2016	27669155	Patients aged between 16 and 70 years with uncontrolled partial-onset seizures, with or without secondary generalization (Commission on Classification and Terminology of the International League Against Epilepsy, 1981), were eligible for study enrollment if they were taking stable daily	Placebo	184	102 (55.4)	31.8 (12.0)	16.8 (11.5)
				Lacosamide 200 mg/day	183	94 (51.4)	33.2 (12.2)	18.3 (10.9)
				Lacosamide 400 mg/day	180	104 (57.8)	32.3 (11.9)	17.9 (11.7)
Inoue	2021	34246118	Male and female aged 16–70 years who completed a double-blind trial	Placebo	164	91 (55.5)	32.2 (12.2)	17.0 (11.6)
				Lacosamide 200 mg/day	163	84 (51.5)	33.6 (12.5)	18.4 (10.8)
				Lacosamide 400 mg/day	146	84 (57.5)	32.2 (11.4)	16.5 (10.7)
Kalviainen	1998	9551842	16–75 years, had a documented history of partial seizures (six in the previous 8 weeks) supported by one of the following findings: an interictal electroencephalogram (EEG) demonstrating a focal abnormality; an interictal EEG demonstrating unilateral or bilateral asynchronous activity; or evidence of a focal CNS lesion by computed tomography o magnetic resonance imaging	Placebo	77	58.4	16–75	23.0 (1–49)
				Tiagabine 30 mg/day	77			24.9 (2–52)
Klein	2015	26471380	Eligible patients were aged ≥16–80 years, with well characterized focal epilepsy or epileptic syndrome	Placebo	261	51%	39.8 (12.5)	22.7 (13.3)
				Brivaracetam 100 mg/day	253	40.3%	39.1 (13.4)	22.2 (13.3)
				Brivaracetam 200 mg/day	250	53.2%	39.8 (12.8)	23.4 (14.6)
Krauss	2020	31734103	Eligible patients were adults aged 18–70 years with a diagnosis of focal epilepsy according to the International League Against Epilepsy’s Classification of Epileptic Seizures. The epilepsy had to be uncontrolled despite treatment with at least one anti-epileptic drug within the past 2 years. Patients must have been taking one to three concomitant ASM at stable doses for at least 4 weeks before screening	Placebo	108	50.5	18–70	NA
				Cenobamate 100 mg/day	108			NA
				Cenobamate 200 mg/day	110			NA
				Cenobamate 400 mg/day	111			NA
Lee	2009	19222545	≥18 years patients were required to have tried at least one ASM at the maximally tolerable dose and had to be taking one to three ASMs at a clinically relevant dose. Additional inclusion criteria included a minimum of four seizures that had occurred over at least 2 days during a 6-week base line period with no 28-day seizure-free period	Placebo	59	58	35.1	18 (0.7–48.1)
				Pregabalin 150–600 mg/day	119	44	33.3	16.5 (0.3–48.0)
Lindberger	2000	11051124	Patients with partial epilepsy were eligible if they had tried no more than two ASM monotherapy regimens	Gabapentin 2,400 and 3,600 mg/day	50	28 (56)	34.5 (13–68)	3.5 (0–36)
				Vigabatrin 2,000 and 4,000 mg/day	52	23 (44)	33 (14–56)	9.5 (0–43)
Matsuo	1993	8232944	Patient population. Men or women, aged 18–65 years (inclusive), were eligible for the study if they demonstrated simple or complex partial seizures (with or without secondary generalization) that were refractory to treatment with up to three currently marketed ASMs	Placebo	73	22 (30%)	34 (18–63)	21.5
				Lamotrigine 300 mg/day	71	30 (42%)	33 (20–57)	22.4
				Lamotrigine 500 mg/day	72	15 (12%)	32 (18–59)	21.8
Naritoku	2007	17938371	Patients more than 12 years old diagnosed with epilepsy with partial seizures and taking one to two baseline ASM were randomized to adjunctive once-daily lamotrigine or placebo in a double-blind, parallel-group trial	Placebo	121	49.6	≧12	22.1 ± 16.1
				Lamotrigine 200/300/500 mg/day	118			21.8 ± 13.2
Nishida	2018	29250772	Eligible patients were aged ≥12 years; diagnosed with partial-onset seizures, with or without SG seizures, according to the 1981 International League Against Epilepsy Classification of Epileptic Seizures9; had uncontrolled partial-onset seizures, despite ≥2 ASMs within the last 2 years; ≥5 partial-onset seizures during baseline; and were taking stable doses of 1–3 approved concomitant ASMs. Only one ASM was permitted (carbamazepine, phenytoin, or oxcarbazepine)	Placebo	175	86 (49.1)	34.5 (13.2)	17.5 (10.9)
				Perampanel 4 mg/day	174	80 (46)	33.1 (13.2)	17.4 (11.1)
				Perampanel 8 mg/day	175	91 (52)	33.6 (14.1)	16.9 (11.5)
				Perampanel 12 mg/day	180	87 (48.3)	32.3 (12.3)	17.4 (11.2)
No authors listed	1993	8232945	≥16 years, only patients with documented partial seizures refractory to treatment with currently available ASMs were enrolled in the study. To qualify, patients had to have had an average of at least four clearly recognizable partial seizures per month for the 3 months prior to baseline, while taking one or two ASMs at stable dosages	Placebo	95	69 (70)	34 (17–66)	22 (2–49)
				Gabapentin 600 mg/day	49	36 (68)	34 (16–67)	20 (3–36)
				Gabapentin 1,200 mg/day	91	60 (59)	35 (19–65)	21 (3–45)
				Gabapentin 1,800 mg/day	53	37 (69)	35 (18–70)	21 (1–41)
No authors listed	1990	1971862	Patients eligible for the study were those with at least 1 partial seizure per week, with or without secondary generalization, despite adequate medication with one or two standard anticonvulsants	Placebo	66	41.7	14–73	17 (2–47)
				Gabapentin 1,200 mg/day	61			19 (4–38)
Peltola	2009	19317886	12–70 years of, with recurrent partial-onset seizures despite receiving at least one but no more than three concomitant ASMs. Weigh ≥50 kg and have a confirmed diagnosis of partial-onset seizures, whether or not secondarily generalized, for at least 6 months preceding the screening visit and refractory to pharmacotherapy with one to three ASMs. During the 8-week baseline period, patients were required to have at least eight partial seizures, with or without secondary generalization, and at least two partial seizures in each 4-week inter	Placebo	79	62.7	12–68	16.43 ± 11.93
				Levetiracetam 1,000 mg/day	79			13.11 ± 10.87
Privitera	1996	8649569	18–65 years with a history of refractory partial epilepsy with or without secondary generalization were eligible for participation in the study	Placebo	47	70.2	35.0 (18–68)	NA
				Topiramate 600 mg/day	48	79.2	35.6 (18–57)	NA
				Topiramate 800 mg/day	48	85.4	34.3 (18–67)	NA
				Topiramate 1,000 mg/day	47	85.1	36.3 (18–64)	NA
Sackellares	2004	15144425	17–65 years patients had to be receiving at least one, but no more than two of the following ASMs, had a history of at least four complex partial seizures per month; and had no more than eight generalized tonics	Placebo	74	58.1	36.4 (17.8–67.5)	NA
				Zonisamide 7 mg/kg/day	78	74.4	35.6 (17.9–64.1)	NA
Schmidt	1993	8325280	18–59 years. During the 4 months preceding the baseline period all patients had an average of at least four complex partial seizures per month in spite of therapeutic plasma concentrations of standard ASM. The diagnosis of seizure types was based on the International Classification of Epileptic Seizures	Placebo	68	139	18–59	23.5
				Zonisamide 500 mg/day	71			20.9
Sharief	1996	8956919	18–65 years have an unequivocal history of partial seizures with or without secondarily generalized seizures. Those patients who had at least 8 partial seizures during an 8-week baseline period in which they were maintained at therapeutic plasma ASM concentrations were qualified to enter the double-blind treatment phase. Patients with a seizure-free interval that exceeded 3 weeks or with more than one seizure-free interval of 3 weeks during the baseline period were excluded	Placebo	24	72.2	32.6	NA
				Topiramate 400 mg/day	23	91.3	35.4	NA
Shorvon	2000	10999557	16–65 years patients were required to maintain stable dose regimens of a maximum of two ASMs for at least 4 weeks before the selection visit, as well as throughout the study. Patients had to have at least four partial seizures during each 4-week interval in the 8- or 12-week baseline period	Placebo	112	49	37 (16–69)	23.2 ± 11.0
				Levetiracetam 1,000 mg/day	106	48	36 (16–68)	23.8 ± 12.3
				Levetiracetam 2,000 mg/day	106	48	37 (14–65)	23.6 ± 13.3
Tassinari	1996	8764816	18–65 years patients who met the requirements for inclusion during the screening phase were further evaluated during an 8-week baseline phase in which they were required to have at least eight partial seizures while being maintained at therapeutic plasma ASM concentrations	Placebo	29	68	32.9	NA
				Topiramate 600 mg/day	26			NA
Tsai	2006	16417534	16–60 years, all randomized patients had been diagnosed as having epilepsy for ≥6 months before the study. Partial seizures were treatment resistant in all cases, and, during an 8-week baseline period, all patients had at least four complex or secondarily generalized partial seizures	Placebo	47	53.2	31.7	18.7 ± 10.7
				Levetiracetam 2000 mg/day	47	36.2	32.8	18.6 ± 8.5
Uthman	1998	9443711	12–77 years, good health except for epilepsy; occurrence of at least 6 CPS alone or in combination with any other seizure type in the 8 weeks preceding the screening visit (with each of the two 4-week segments containing at least l CPS); electroencephalographic evidence of a unilateral or bilateral abnormality consistent with CPS; and availability of at least I neuroimaging study of the brain to rule out the presence of any progressive lesions	Placebo	90	58	12–77	22.9 (1.4–65.8)
				Tiagabine 16 mg/day	61			
				Tiagabine 32 mg/day	86			
				Tiagabine 56 mg/day	55			
Wu	2009	18657175	16–70 years, patients had to present with treatment-resistant partial onset seizures to be eligible and had to have experienced at least eight partial-onset seizures during the 8-week historical baseline period	Placebo	100	54	32.8 (16–64)	17.3 ± 12.1
				Levetiracetam 1,000–3,000 mg/day	102	50	32.7 (15–70)	16.5 ± 12.7
Xiao	2009	19176965	16–70 years were invalid to current anti-epileptic therapy and had experienced at least 4 seizures per month (averaged over the preceding 2 months, despite therapy with other marketed ASMs)	Placebo	28	42.9	32.5 (18–58)	16.1 ± 12.5
				Levetiracetam 3,000 mg/day	28	42.9	32.8 (17–60)	14.1 ± 9.4
Yamauchi	2006	16884455	≥16 years, with partial seizures as defined by criteria developed by the International League Against Epilepsy. Weighing 40–110 kg, were eligible if they were on a stable dose of no more than two ASM.	Placebo	82	48.3	≧16	19.5 (2.1–47.0)
				Gabapentin 1,200 mg/day	86			19.8 (4.0–42.0)
				Gabapentin 1800 mg/day	41			21.2 (5.2–43.3)
Yen	2000	10999555	18–65 years of age with a history of partial seizures that had not responded to adequate doses of ASM treatment for 2 or more years	Placebo	23	56.5	32.0 (22–48)	18.9 ± 11.1
				Topiramate 300 mg/day	23	26.1	31.4 (18–54)	14.9 ± 10.9
Zaccara	2014	24902473	Patients were aged ≥18 years, with a diagnosis of epilepsy with partial seizures (equivalent to focal seizures in the 2010 ILAE classification), which were historically inadequately controlled with at least 2, but no more than 5, prior ASMs	Pregabalin150, 300, 450 and 600 mg/day	254	120 (47.2)	32.7 ± 11.2	15.5 (2.0–52.8
				Levetiracetam 1,000, 2000 and 3,000 mg/day	255	125 (49.0)	36.3 ± 12.2	17.3 (1.9–59.6)
Zhou	2008	18024209	16–70 years, whose partial-onset seizures (simple or complex partial with or without secondary generation, according to the International League Against Epilepsy classification) were poorly controlled by at least one first-line ASM at the time of the study. Poor control was defined as having a minimum of eight seizures during the 8-week baseline period with a minimum of two seizures during each 4-week period	Placebo	11	54.1	16–70	16.5 ± 7.2
				Levetiracetam 3,000 mg/day	13			8.7 ± 6.4

Note: ASMs: Anti-seizure medications; CNS: central nervous system; CP: complex partial; CPS: complex partial seizures; CT: computed tomography; EEG: electroencephalo-graph; ILAE: International League Against Epilepsy; MRI: magnetic resonance imaging; NA: no reported; SGS: secondary generalization; SP: simple partial; VNS: vagus nerve stimulation.

### Effective outcome

#### 50% Response rate

**FIGURE 2:**
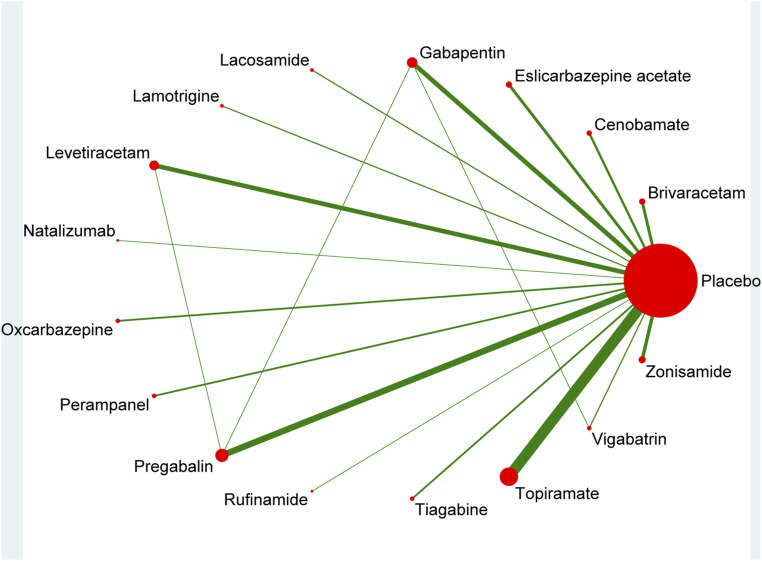
Network plot for 50% response rate.

The pool of 46 RCTs (Gabapentin in Partial Epilepsy, 1990; Anhut et al., 1994; The US Gabapentin Study Group No. 5, 1993; Schmidt et al., 1993; French et al., 1996; Privitera et al., 1996; Faught et al., 1996; Tassinari et al., 1996; Sharief et al., 1996; Faught, 1997; Ben-Menachem, 1997; Uthman et al., 1998; Bruni et al., 2000; Cereghino et al., 2000; Yen et al., 2000; Shorvon et al., 2000; Lindberger et al., 2000; Barcs et al., 2000; French et al., 2003; Arroyo et al., 2004; Sackellares et al., 2004; Brodie, 2004; Brodie et al., 2005; Beydoun et al., 2005; Tsai et al., 2006; Yamauchi et al., 2006; Naritoku et al., 2007; Zhou et al., 2008; Wu et al., 2009; Xiao et al., 2009; Lee et al., 2009; Gil-Nagel et al., 2009; Ben-Menachem et al., 2010; French et al., 2010; Biton et al., 2011; Zaccara et al., 2014; French et al., 2014; Hogan et al., 2014; Klein et al., 2015; French et al., 2016; Hong et al., 2016; Nishida et al., 2018; Krauss et al., 2020; Chung et al., 2020; French et al., 2021; Baulac et al., 2010), including 12,120 study participants, contributed to the analysis of the 50% response rate. Figure 2 illustrated a network plot of 50% response rate assessment of 16 eligible ASMs as adjunctive therapy and placebo for the treatment of drug-resistant focal epilepsy.

As shown in Table 2, the consequence of direct comparisons showed that the following ASMs as adjunctive therapy, including brivaracetam, cenobamate, eslicarbazepine acetate, gabapentin, lacosamide, levetiracetam, oxcarbazepine, perampanel, pregabalin, rufinamide, tiagabine, topiramate, vigabatrin and zonisamide, demonstrated statistically significant in 50% response rate than that of placebo. Nevertheless, the other results were no statistically significant differences.

**TABLE 2:** Results of network and traditional paired meta-analysis for 50% response rate.

Placebo	2.07 (1.53, 2.81)	2.12 (1.56, 2.88)	1.95 (1.41, 2.70)	2.30 (1.76, 3.02)	2.22 (1.47, 3.35)	1.55 (1.00, 2.40)	2.43 (1.88, 3.15)	1.77 (0.65, 4.86)	3.03 (2.08, 4.40)	1.72 (1.21, 2.44)	2.06 (1.70, 2.50)	2.28 (1.20, 4.31)	4.07 (2.03, 8.18)	3.10 (2.44, 3.95)	2.34 (1.58, 3.46)	2.40 (1.76, 3.27)
**1.94 (1.60, 2.36)**	Brivaracetam	1.02 (0.67, 1.58)	0.94 (0.60, 1.47)	1.11 (0.74, 1.67)	1.07 (0.64, 1.79)	0.75 (0.44, 1.28)	1.18 (0.79, 1.75)	0.86 (0.30, 2.45)	1.46 (0.90, 2.37)	0.83 (0.52, 1.32)	0.99 (0.70, 1.42)	1.10 (0.54, 2.23)	1.97 (0.92, 4.21)	**1.50 (1.02, 2.21)**	1.13 (0.69, 1.85)	1.16 (0.75, 1.79)
**2.14 (1.76, 2.59)**	/	Cenobamate	0.92 (0.59, 1.44)	1.09 (0.72, 1.63)	1.04 (0.62, 1.75)	0.73 (0.43, 1.25)	1.15 (0.77, 1.71)	0.83 (0.29, 2.40)	1.43 (0.88, 2.31)	0.81 (0.51, 1.29)	0.97 (0.67, 1.39)	1.07 (0.53, 2.18)	1.92 (0.90, 4.11)	1.46 (0.99, 2.16)	1.10 (0.67, 1.81)	1.13 (0.73, 1.75)
**1.95 (1.41, 2.71)**	/	/	Eslicarbazepine acetate	1.18 (0.77, 1.80)	1.14 (0.67, 1.92)	0.80 (0.46, 1.37)	1.25 (0.82, 1.89)	0.91 (0.31, 2.62)	1.55 (0.95, 2.54)	0.88 (0.55, 1.42)	1.05 (0.72, 1.54)	1.17 (0.57, 2.39)	2.09 (0.97, 4.50)	**1.59 (1.06, 2.38)**	1.20 (0.72, 1.99)	1.23 (0.78, 1.92)
**2.52 (1.89, 3.37)**	/	/	/	Gabapentin	0.96 (0.59, 1.58)	0.67 (0.40, 1.13)	1.06 (0.73, 1.52)	0.77 (0.27, 2.19)	1.31 (0.83, 2.08)	0.75 (0.48, 1.16)	0.89 (0.66, 1.20)	0.99 (0.49, 1.98)	1.77 (0.84, 3.74)	1.35 (0.94, 1.93)	1.02 (0.68, 1.52)	1.04 (0.69, 1.57)
**2.22 (1.75, 2.83)**	/	/	/	/	Lacosamide	0.70 (0.38, 1.28)	1.10 (0.67, 1.79)	0.80 (0.27, 2.38)	1.37 (0.78, 2.38)	0.78 (0.45, 1.33)	0.93 (0.59, 1.47)	1.03 (0.48, 2.20)	1.84 (0.82, 4.13)	1.40 (0.87, 2.26)	1.06 (0.60, 1.86)	1.08 (0.65, 1.81)
1.54 (0.86, 2.74)	/	/	/	/	/	Lamotrigine	1.57 (0.94, 2.60)	1.14 (0.38, 3.42)	**1.95 (1.10, 3.46)**	1.11 (0.64, 1.94)	1.33 (0.82, 2.14)	1.47 (0.68, 3.18)	**2.62 (1.15, 5.97)**	**2.00 (1.21, 3.29)**	1.51 (0.84, 2.70)	1.54 (0.91, 2.63)
**2.57 (1.93, 3.42)**	/	/	/	/	/	/	Levetiracetam	0.73 (0.26, 2.06)	1.24 (0.79, 1.96)	0.71 (0.46, 1.09)	0.85 (0.63, 1.13)	0.94 (0.47, 1.86)	1.67 (0.80, 3.52)	1.28 (0.90, 1.81)	0.96 (0.60, 1.53)	0.99 (0.66, 1.48)
1.77 (0.73, 4.31)	/	/	/	/	/	/	/	Natalizumab	1.71 (0.58, 5.01)	0.97 (0.33, 2.83)	1.16 (0.42, 3.25)	1.28 (0.39, 4.24)	2.30 (0.67, 7.84)	1.75 (0.62, 4.95)	1.32 (0.45, 3.90)	1.35 (0.47, 3.89)
**3.03 (2.13, 4.32)**	/	/	/	/	/	/	/	/	0xcarbazepine	**0.57 (0.34, 0.95)**	0.68 (0.45, 1.04)	0.75 (0.36, 1.58)	1.35 (0.61, 2.97)	1.03 (0.66, 1.60)	0.77 (0.45, 1.33)	0.79 (0.49, 1.29)
**1.72 (1.21, 2.46)**	/	/	/	/	/	/	/	/	/	Perampanel	1.20 (0.80, 1.78)	1.32 (0.64, 2.74)	**2.37 (1.08, 5.16)**	**1.80 (1.18, 2.75)**	1.36 (0.81, 2.29)	1.39 (0.87, 2.22)
**2.10 (1.51, 2.94)**	/	/	/	0.96 (0.82, 1.12)	/	/	1.007 (0.84, 1.20)	/	/	/	Pregabalin	1.11 (0.57, 2.16)	1.98 (0.96, 4.08)	**1.51 (1.11, 2.05)**	1.14 (0.74, 1.74)	1.16 (0.81, 1.68)
**2.28 (1.49, 3.48)**	/	/	/	/	/	/	/	/	/	/	/	Rufinamide	1.79 (0.69, 4.61)	1.36 (0.69, 2.70)	1.03 (0.49, 2.18)	1.05 (0.52, 2.14)
**4.08 (2.05, 8.12)**	/	/	/	/	/	/	/	/	/	/	/	/	Tiagabine	0.76 (0.36, 1.59)	0.57 (0.26, 1.28)	0.59 (0.27, 1.26)
**2.99 (2.43, 3.68)**	/	/	/	/	/	/	/	/	/	/	/	/	/	Topiramate	0.75 (0.48, 1.19)	0.77 (0.52, 1.14)
**2.07 (1.45, 2.95)**	/	/	/	1.21 (0.88, 1.67)	/	/	/	/	/	/	/	/	/	/	Vigabatrin	1.02 (0.62, 1.69)
**2.43 (1.93, 3.06)**	/	/	/	/	/	/	/	/	/	/	/	/	/	/	/	Zonisamide

Note: Comparisons between anti-seizure medications should be read from right to left, and the results are all comparisons between treatments defined on the bottom right and treatments defined on the top left. The table is divided into lower left and upper right sections with anti-seizure medications as the dividing line. The upper right represents the network comparison results, and the lower left part represents the direct comparison results. For comparison results, when relative risk (RR) < 1, tended to define treatment on the left, when RR > 1, treatment tends to be defined to the right. Significant results are in bold and underline, and “/” means that the results are not available.

Compared with placebo in the network meta-analysis, ASMs as adjunctive therapy, including brivaracetam, cenobamate, eslicarbazepine acetate, gabapentin, lacosamide, lamotrigine, levetiracetam, oxcarbazepine, perampanel, pregabalin, rufinamide, tiagabine, topiramate, vigabatrin, and zonisamide, demonstrated statistically significant in 50% response rate, as detailed in Table 2. The results of other ASMs as adjunctive therapy were shown in Table 2.

**FIGURE 3:**
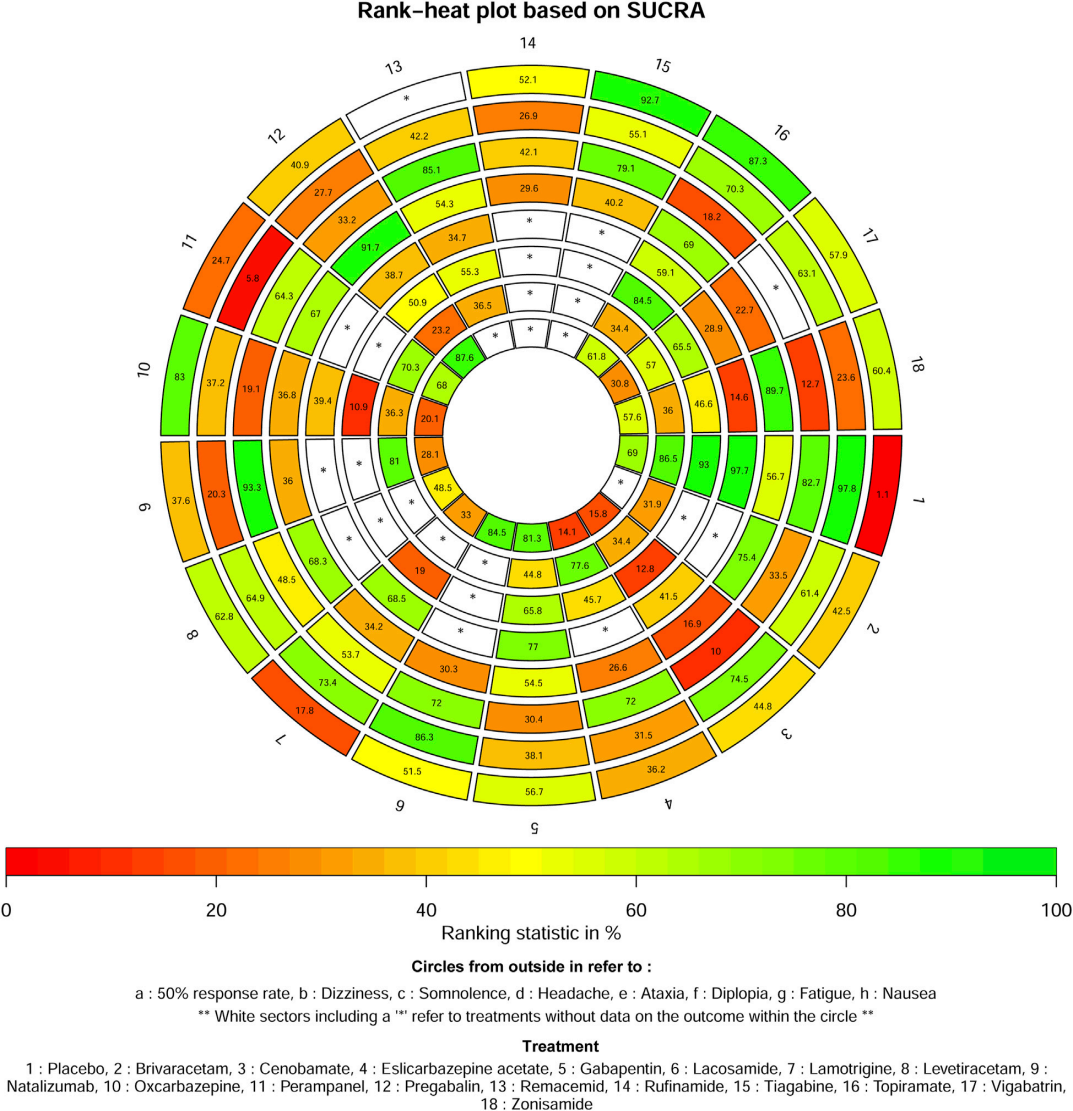
Ranking for all outcomes.

The ASMs as adjunctive therapy were assessed and graded based on the SUCRA, with tiagabine (92.7%) demonstrating the most optimal therapeutic outcome, subsequent to topiramate (87.3%), oxcarbazepine (83%) and levetiracetam (62.8%). The three ASMs as adjunctive therapy with the worst therapeutic effects were placebo (1.1%), lamotrigine (17.8%) and perampanel (24.7%) in Figure 3.

### Safety outcomes

#### Dizziness

A total of 45 studies (Gabapentin in Partial Epilepsy, 1990; Anhut et al., 1994; The US Gabapentin Study Group No. 5, 1993; Schmidt et al., 1993; Privitera et al., 1996; Faught et al., 1996; Tassinari et al., 1996; Uthman et al., 1998; Bruni et al., 2000; Cereghino et al., 2000; Yen et al., 2000; Shorvon et al., 2000; Barcs et al., 2000; French et al., 2003; Arroyo et al., 2004; Brodie, 2004; Brodie et al., 2005; Beydoun et al., 2005; Tsai et al., 2006; Yamauchi et al., 2006; Naritoku et al., 2007; Wu et al., 2009; Xiao et al., 2009; Lee et al., 2009; Gil-Nagel et al., 2009; Ben-Menachem et al., 2010; French et al., 2010; Biton et al., 2011; Zaccara et al., 2014; French et al., 2014; Klein et al., 2015; French et al., 2016; Hong et al., 2016; Nishida et al., 2018; Krauss et al., 2020; Chung et al., 2020; French et al., 2021; Baulac et al., 2010; Matsuo et al., 1993; Ben-Menachem et al., 1996; Kälviäinen et al., 1998; Chadwick et al., 2000; Guberman et al., 2002; Peltola et al., 2009; Inoue et al., 2021) comprising 12,608 participants contributed to the analysis of the safety outcome of dizziness. Supplementary Figure S1 illustrated a network plot of the safety outcomes dizziness assessment of 17 eligible ASMs as adjunctive therapy and placebo for the treatment of drug-resistant focal epilepsy.

As shown in Supplementary Table S2, the consequence of direct comparisons showed that, compared with placebo, the following ASMs as adjunctive therapy demonstrated statistically significant in dizziness: cenobamate, eslicarbazepine acetate, gabapentin, levetiracetam, oxcarbazepine, perampanel, pregabalin, remacemid, rufinamide, tiagabine, topiramate and zonisamide. Nevertheless, the other results were no statistically significant differences.

The findings of the network meta-analysis indicated that, compared with placebo, ASMs as adjunctive therapy, including brivaracetam, cenobamate, eslicarbazepine acetate, gabapentin, lamotrigine, levetiracetam, oxcarbazepine, perampanel, pregabalin, remacemid, rufinamide, tiagabine, topiramate and zonisamide, demonstrated statistically significant in dizziness (Supplementary Table S2). The results of other ASMs as adjunctive therapy were shown in Supplementary Table S2.

According to the SUCRA, all ASMs as adjunctive therapy assessed for the safety outcome of dizziness were rated, with placebo (97.8%) exhibiting the best therapeutic benefit, subsequently followed by lacosamide (86.3%), cenobamate (74.5%) and lamotrigine (73.4%). The three ASMs as adjunctive therapy with the worst therapeutic effects were perampanel (5.8%), natalizumab (20.3%) and zonisamide (23.6%) (Figure 3).

#### Somnolence

A total of 42 studies (Gabapentin in Partial Epilepsy, 1990; Anhut et al., 1994; The US Gabapentin Study Group No. 5, 1993; Schmidt et al., 1993; Privitera et al., 1996; Faught et al., 1996; Tassinari et al., 1996; Sharief et al., 1996; Cereghino et al., 2000; Shorvon et al., 2000; Barcs et al., 2000; French et al., 2003; Arroyo et al., 2004; Brodie, 2004; Brodie et al., 2005; Beydoun et al., 2005; Tsai et al., 2006; Yamauchi et al., 2006; Naritoku et al., 2007; Wu et al., 2009; Xiao et al., 2009; Lee et al., 2009; Gil-Nagel et al., 2009; Ben-Menachem et al., 2010; French et al., 2010; Biton et al., 2011; Zaccara et al., 2014; French et al., 2014; Klein et al., 2015; French et al., 2016; Hong et al., 2016; Nishida et al., 2018; Krauss et al., 2020; Chung et al., 2020; French et al., 2021; Baulac et al., 2010; Matsuo et al., 1993; Kälviäinen et al., 1998; Chadwick et al., 2000; Guberman et al., 2002; Peltola et al., 2009; Inoue et al., 2021) encompassing 12,163 participants contributed to the analysis of the safety outcome of somnolence. Supplementary Figure S2 illustrated a network plot of safety outcomes somnolence assessment of 16 eligible ASMs as adjunctive therapy and placebo for the treatment of drug-resistant focal epilepsy.

In the results of direct comparisons, compared with placebo, ASMs as adjunctive therapy including cenobamate, gabapentin, levetiracetam, oxcarbazepine, pregabalin, topiramate and zonisamide demonstrated statistically significant in somnolence (Supplementary Table S3). Nevertheless, the other results were no statistically significant differences.

The findings of the network meta-analysis indicated that, compared with placebo, ASMs as adjunctive therapy, including brivaracetam, cenobamate, gabapentin, levetiracetam, oxcarbazepine, pregabalin, topiramate and zonisamide, demonstrated statistically significant in somnolence (Supplementary Table S3). The results of other ASMs as adjunctive therapy were shown in Supplementary Table S3.

The ASMs as adjunctive therapy were ranked based on the SUCRA and the results indicate that natalizumab (93.3%) exhibited the most favourable therapeutic effect, subsequent to remacemide (85.1%), placebo (82.7%) and tiagabine (79.1%). The three ASMs as adjunctive therapy with the worst therapeutic effects were cenobamate (10%), zonisamide (12.7%) and topiramate (18.2%) in Figure 3.

#### Headache

A total of 38 studies (Anhut et al., 1994; The US Gabapentin Study Group No. 5, 1993; Privitera et al., 1996; Faught et al., 1996; Tassinari et al., 1996; Sharief et al., 1996; Bruni et al., 2000; Cereghino et al., 2000; Yen et al., 2000; Shorvon et al., 2000; Barcs et al., 2000; French et al., 2003; Arroyo et al., 2004; Brodie et al., 2005; Tsai et al., 2006; Yamauchi et al., 2006; Naritoku et al., 2007; Wu et al., 2009; Lee et al., 2009; Gil-Nagel et al., 2009; Ben-Menachem et al., 2010; French et al., 2010; Biton et al., 2011; Zaccara et al., 2014; Klein et al., 2015; French et al., 2016; Hong et al., 2016; Nishida et al., 2018; Krauss et al., 2020; Chung et al., 2020; French et al., 2021; Baulac et al., 2010; Matsuo et al., 1993; Ben-Menachem et al., 1996; Kälviäinen et al., 1998; Chadwick et al., 2000; Peltola et al., 2009; Inoue et al., 2021) encompassing 11,011 participants contributed to the analysis of the headache safety outcome. Supplementary Figure S3 illustrated a network plot of the safety outcomes headache assessment of 17 eligible ASMs as adjunctive therapy and placebo for the treatment of drug-resistant focal epilepsy.

In the results of direct comparisons, compared with placebo, ASMs as adjunctive therapy including pregabalin, demonstrated statistically significant in headache (Supplementary Table S4). Nevertheless, the other results were no statistically significant differences.

The findings of the network meta-analysis indicated that, compared with placebo, ASMs as adjunctive therapy, including pregabalin, demonstrated statistically significant in headache (Supplementary Table S4). The results of other ASMs as adjunctive therapy were shown in Supplementary Table S4.

The ASMs as adjunctive therapy were ranked based on the SUCRA, with pregabalin (91.7%) showing the best therapeutic effect, subsequent to zonisamide (89.7%), brivaracetam (75.4%) and topiramate (69%). The three ASMs as adjunctive therapy exhibiting the most unfavorable therapeutic effects were cenobamate (16.9%), vigabatrin (22.7%) and eslicarbazepine acetate (26.6%) in Figure 3.

#### Ataxia

12 studies (Anhut et al., 1994; The US Gabapentin Study Group No. 5, 1993; Faught et al., 1996; Bruni et al., 2000; Barcs et al., 2000; French et al., 2003; Brodie, 2004; Beydoun et al., 2005; Krauss et al., 2020; Baulac et al., 2010; Matsuo et al., 1993; Chadwick et al., 2000) encompassing 3,596 study participants contributed to the analysis of the safety outcome of ataxia. Supplementary Figure S4 illustrated a network plot of safety outcomes ataxia assessment of 9 eligible ASMs as adjunctive therapy and placebo for the treatment of drug-resistant focal epilepsy.

In the results of direct comparisons, compared with placebo, ASMs as adjunctive therapy including cenobamate, gabapentin, oxcarbazepine, pregabalin, topiramate, zonisamide, demonstrated statistically significant in ataxia (Supplementary Table S5). Nevertheless, the other results were no statistically significant differences.

The findings of the network meta-analysis indicated that, compared with placebo, ASMs as adjunctive therapy, including cenobamate, gabapentin, lamotrigine, oxcarbazepine, pregabalin, topiramate, zonisamide, demonstrated statistically significant in ataxia (Supplementary Table S5). The results of other ASMs as adjunctive therapy were shown in Supplementary Table S5.

The ASMs as adjunctive therapy were ranked based on the SUCRA, with the placebo (97.7%) demonstrating optimal therapeutic effectiveness, subsequent to gabapentin (77%) and lamotrigine (68.5%). The three ASMs as adjunctive therapy with the worst therapeutic effects were zonisamide (14.6%), vigabatrin (28.9%) and remacemide (34.7%) in Figure 3.

#### Diplopia

The safety outcome study of diplopia included 16 studies (Anhut et al., 1994; The US Gabapentin Study Group No. 5, 1993; Privitera et al., 1996; Faught et al., 1996; Bruni et al., 2000; Barcs et al., 2000; Arroyo et al., 2004; Beydoun et al., 2005; Yamauchi et al., 2006; Gil-Nagel et al., 2009; Ben-Menachem et al., 2010; Krauss et al., 2020; Baulac et al., 2010; Matsuo et al., 1993; Kälviäinen et al., 1998; Chadwick et al., 2000) with 4,487 participants. Supplementary Figure S5 illustrated a network plot of the safety outcomes diplopia assessment of 10 eligible ASMs as adjunctive therapy and placebo for the treatment of drug-resistant focal epilepsy.

In the results of direct comparisons, compared with placebo, ASMs as adjunctive therapy including oxcarbazepine cenobamate, eslicarbazepine acetate, gabapentin, lamotrigine, oxcarbazepine, pregabalin and topiramate, demonstrated statistically significant in diplopia (Supplementary Table S6). Nevertheless, the other results were no statistically significant differences.

The findings of the network meta-analysis indicated that, compared with placebo, ASMs as adjunctive therapy, including cenobamate, eslicarbazepine acetate, gabapentin, lamotrigine, oxcarbazepine, pregabalin and topiramate, demonstrated statistically significant in diplopia (Supplementary Table S6). The results of other ASMs as adjunctive therapy were shown in Supplementary Table S6.

The ASMs as adjunctive therapy were ranked based on the SUCRA, with the placebo (93%) demonstrating optimal therapeutic effectiveness, subsequent to topiramate (84.5%) and gabapentin (65.8%). The three ASMs as adjunctive therapy with the worst therapeutic effects were oxcarbazepine (10.9%), cenobamate (12.8%) and lamotrigine (19%) in Figure 3.

#### Fatigue

A total of 22 studies (Anhut et al., 1994; The US Gabapentin Study Group No. 5, 1993; Schmidt et al., 1993; Privitera et al., 1996; Faught et al., 1996; Tassinari et al., 1996; Sharief et al., 1996; Bruni et al., 2000; Barcs et al., 2000; Brodie, 2004; Lee et al., 2009; Ben-Menachem et al., 2010; French et al., 2010; French et al., 2014; Klein et al., 2015; Nishida et al., 2018; Krauss et al., 2020; Chung et al., 2020; French et al., 2021; Ben-Menachem et al., 1996; Chadwick et al., 2000; Guberman et al., 2002) comprising 5,800 participants contributed to the analysis of the safety outcome of fatigue. Supplementary Figure S6 illustrated a network plot of the safety outcomes fatigue assessment of 12 eligible ASMs as adjunctive therapy and placebo for the treatment of drug-resistant focal epilepsy.

In the results of direct comparisons, compared with placebo, ASMs as adjunctive therapy including brivaracetam, cenobamate, gabapentin, oxcarbazepine, topiramate and zonisamide, demonstrated statistically significant in fatigue (Supplementary Table S7). Nevertheless, the other results were no statistically significant differences.

The findings of the network meta-analysis indicated that, compared with placebo, ASMs as adjunctive therapy, including brivaracetam, cenobamate, gabapentin, oxcarbazepine, topiramate, and zonisamide, demonstrated statistically significant in fatigue (Supplementary Table S7). The results of other ASMs as adjunctive therapy were shown in Supplementary Table S7.

The ASMs as adjunctive therapy were ranked based on the SUCRA, with the placebo (86.5%) demonstrating optimal therapeutic effectiveness, subsequent to natalizumab (81%) and eslicarbazepine acetate (77.6%). The three ASMs as adjunctive therapy with the worst therapeutic effects were pregabalin (23.2%), brivaracetam (31.9%) and cenobamate (34.4%), in Figure 3.

#### Nausea

A total of 21 studies (Anhut et al., 1994; Schmidt et al., 1993; Tassinari et al., 1996; Yen et al., 2000; Shorvon et al., 2000; Barcs et al., 2000; Brodie et al., 2005; Yamauchi et al., 2006; Naritoku et al., 2007; Gil-Nagel et al., 2009; Ben-Menachem et al., 2010; Zaccara et al., 2014; French et al., 2014; Nishida et al., 2018; Krauss et al., 2020; Chung et al., 2020; French et al., 2021; Matsuo et al., 1993; Kälviäinen et al., 1998; Peltola et al., 2009; Inoue et al., 2021) encompassing 6,235 participants contributed to the safety outcome of nausea. Supplementary Figure S7 illustrated a network plot of the safety outcomes nausea assessment of 13 eligible ASMs as adjunctive therapy and placebo for the treatment of drug-resistant focal epilepsy.

In the results of direct comparisons, compared with placebo, ASMs as adjunctive therapy, including lamotrigine and oxcarbazepine demonstrated statistically significant in nausea (Supplementary Table S8). Nevertheless, the other results were no statistically significant differences.

The findings of the network meta-analysis indicated that, compared with placebo, ASMs as adjunctive therapy, cenobamate, eslicarbazepine acetate, lamotrigine and oxcarbazepine demonstrated statistically significant in nausea (Supplementary Table S8). In addition, except for a limited number of combination comparisons between active ASMs as adjunctive therapy and placebo, no statistically significant differences were found for the remaining comparisons between active ASMs as adjunctive therapy and placebo in Supplementary Table S8. The results of other ASMs as adjunctive therapy were shown in Supplementary Table S8.

The ASMs as adjunctive therapy were ranked based on the SUCRA, with pregabalin (87.6%) demonstrating optimal therapeutic effectiveness, subsequent to lacosamide (84.5%) and gabapentin (81.3%). The three ASMs as adjunctive therapy exhibiting the worst therapeutic outcomes were eslicarbazepine acetate (14.1%), cenobamate (15.8%) and oxcarbazepine (20.1%) in Figure 3.

#### Test of inconsistency

Since closed loops were not formed for the outcomes of ataxia, fatigue, and diplopia, it was not possible to assess the inconsistency of these loops. Additionally, closed-loop structures were identified for the outcomes of a 50% response rate and adverse events (including dizziness, somnolence, headache, and nausea), and rigorous loop-consistency evaluation revealed no detectable inconsistencies within these loops.

#### Publication bias

No publication bias were revealed in the network funnel plot of all outcomes (Supplementary Figures S8–S15).

## Discussion

While ASMs as adjunctive therapy remained the primary approach for managing epilepsy, some drugs inevitably caused varying degrees of harm to patients. Therefore, physicians must meticulously select specific drugs for treating epilepsy (Iyer and Marson, 2014). The study conducted an evidence-based assessment of comparative effectiveness and safety of ASMs as adjunctive therapy in drug-resistant focal epilepsy. The pertinent findings were as follows: tiagabine, topiramate, zonisamide, levetiracetam, rufinamide, and oxcarbazepine were more effective in controlling seizure frequency (as assessed by seizure-free analysis), whereas lacosamide was less effective than all other ASMs when used as adjunctive therapy.

Tiagabine was mechanistically one of the most precise ASMs in clinical use, but its use was limited to adjunctive therapy for partial seizures with or without secondary generalization in adolescents and adults (Mengel, 1994). Studies had demonstrated that adding tiagabine can reduce the frequency of seizures in individuals with drug-resistant focal seizures (Bresnahan et al., 2019). Another study found that, in the study population, short-term treatment with tiagabine at low doses had no cognitive or electroencephalogram adverse effects compared to placebo. Furthermore, tiagabine therapy did not result in worsening of cognitive function when used at high doses during long-term follow-up (Kälviäinen et al., 1996). Similarly, this study confirmed the substantial superiority of tiagabine in terms of therapeutic effectiveness.

Notably, in this study, topiramate achieved a high ranking for this outcome in 50% response rate (SUCRA: 87.3%), suggesting it may be a favorable first-choice option for this particular outcome. Furthermore, despite the risk of adverse events, such as dizziness, headache, ataxia, and diplopia, topiramate, demonstrated the highest safety profile and the lowest incidence of these events. One study found that when used in the management of drug-resistant focal epilepsy, topiramate could reduce the intensity and frequency of seizures while promoting overall stability, making it an effective, safe, and well-tolerated option for controlling disease progression (Viteva and Zahariev, 2020).

In the current study, levetiracetam exhibited an effective of 50% response rate and a relatively low risk profile (Marson et al., 2021). One study indicated that patients treated with levetiracetam were more prone to experiencing nausea (Zhao et al., 2017). Although levetiracetam lacked approval from the Food and Drug Administration (FDA) as a standalone treatment, it had been frequently used as a first-line ASM in the United States for both focal and generalized tonic-clonic seizures, and as an initial monotherapy in Europe (Abou-Khalil, 2019). Levetiracetam had minimal drug interactions and can be considered as the drug of choice for elderly individuals and fertile women (Sen et al., 2024). However, the findings also revealed that severe psychiatric symptoms, such as anger, violence, and even suicidal thoughts, may occur with levetiracetam administration. In most cases, these mental symptoms can be alleviated or disappear after reducing the dose or discontinuing the drug, but some patients may experience severe mental conditions that negatively impact their quality of life (Tao et al., 2024).

Gabapentin had proven effective as an adjunctive treatment for individuals with drug-resistant focal epilepsy and was generally well-tolerated. However, its used during pregnancy may pose risks to fetal neurodevelopment and congenital malformations (Honybun et al., 2024; Christensen et al., 2024). Some studies (Nakajima-Ohyama et al., 2024) had suggested that gabapentin can improve delirium and serve as a safe alternative therapy, but dose adjustments may be necessary to prevent sleepiness. It is important to note that gabapentin was associated with a higher incidence of dizziness, fatigue, and somnolence compared to placebo (Panebianco et al., 2021), and clinicians and patients should be vigilant of these symptoms during its use.

Pregabalin had demonstrated significant effectiveness in reducing the frequency of seizures in adults with drug-resistant focal epilepsy, but it also carried adverse reactions such as ataxia, dizziness, nausea, and weight gain (Panebianco et al., 2022). When combined with zonisamide, pregabalin had achieved impressive and sustained seizure control in patients with drug-resistant focal epilepsy, with minimal complications and fully reversible effects (Taghdiri et al., 2015).

Oxcarbazepine was an oral medication utilized for the treatment of focal-onset epilepsy, serving both as a monotherapy and an adjunctive therapy (Beydoun et al., 2020). Notably, other studies had indicated that oxcarbazepine exhibited superior overall effectiveness and was associated with fewer adverse events, such as vomiting, compared to other treatments (Zhang et al., 2022). However, it was crucial to acknowledge that our study included relatively small sample sizes for each drug, which may have introduced potential biases in the results. Consequently, further research was required to comprehensively evaluate the effectiveness and safety of oxcarbazepine.

Zonisamide, due to its adverse effects, was unlikely to emerge as the first-line treatment for focal epilepsy (Reimers and Ljung, 2019). Among other treatment options, brivaracetam, considered the second generation of levetiracetam, was a new ASM (Verrotti et al., 2021) that demonstrated high tolerability and effectiveness, particularly for adults with drug-resistant focal epilepsy (Bresnahan et al., 2022). Nevertheless, contrary to preclinical studies suggesting brivaracetam’s potential as an ideal treatment for focal epilepsy (Russo et al., 2017), this study found that the ASM was less effective in practical applications.

Monotherapy was widely accepted as the conventional primary treatment approach for epilepsy. However, when the initial administration of ASMs as adjunctive therapy proved ineffective, the option of employing combination therapy was contemplated. In cases where monotherapy was not controlled, the combination of lamotrigine and levetiracetam was considered. This combination regimen had the highest rate of seizure freedom both before and during pregnancy. Although the effectiveness of either ASM as adjunctive therapy alone may have been similar to that of sodium valproate in the treatment of generalized epilepsy, combination therapy with multiple agents was believed to have better effectiveness (Cohen et al., 2024). For patients who failed to respond to dual therapy, the prognosis could be improved through the reasonable selection of triple therapy, with about 15% of patients with refractory focal epilepsy achieving seizure-free status under triple therapy (Cai et al., 2024).

As indicated in clinical guidelines (Kanner et al., 2018), the following medications were effective in reducing the frequency of treatment-resistant adult focal epilepsy (Level A): immediate-release pregabalin, perampanel, and vigabatrin (though vigabatrin was not considered a first-line treatment). Medications that could reduce the frequency of treatment-resistant adult focal epilepsy (Level B) included lacosamide, eslicarbazepine, extended-release topiramate, and levetiracetam (used as add-on therapy for treatment-resistant childhood focal epilepsy). Perampanel and vigabatrin were found to be effective as add-on treatments for intractable focal epilepsy in adults, whereas oxcarbazepine required a high dose and its efficacy was dose-dependent. The drugs recommended in this study differed from those in the guidelines for several reasons. Firstly, the overall population studied varied, including differences in age and the severity of epilepsy. Secondly, the underlying anti-epileptic medication regimen was unclear. Thirdly, there may have been variations in the amount of adjuvant therapy used across different studies. Fourthly, the quality of research evidence varied across studies. Finally, the small sample size may have affected the accuracy of the results. By expanding the discussion of clinical implications, this study provides a broader and more specific analysis of controversial drugs from previous meta-analyses, making our findings more actionable and relevant to clinicians and patients. This will help ensure that our study has a meaningful impact on the management of drug-refractory focal epilepsy and ultimately improves patient outcomes.

This study had several limitations. Firstly, it lacked sufficient data and subgroup analyses regarding the ethnicity and comorbidities of the participants, which could have substantially impacted the overall conclusion. Secondly, the route of administration may have influenced the potential for side effects associated with each medication, dose, and treatment duration, potentially leading to significant differences among the studies included. Thirdly, we did not evaluate the etiology of drug resistance in drug-resistant focal epilepsy. Fourthly, patient heterogeneity, such as age and comorbidities, was not discussed, which could affect the generalizability of the findings. Fifthly, because some confounding factors were not mentioned in the original studies, subgroup analyses could not be performed. Finally, due to the lack of other safety data, some adverse event outcomes were excluded from the study for comparison, resulting in incomplete conclusions regarding safety.

## Conclusion

This network meta-analysis provided an overview of the 50% response rate and tolerability of the ASMs used in drug-resistant focal seizures, aiming to offer more authoritative and effective guidance for clinical medication guidelines. The analysis demonstrated that topiramate, tiagabine, oxcarbazepine, and levetiracetam were the four most effective adjuvant treatments for ASMs. However, it was important to note that topiramate and oxcarbazepine were associated with a higher risk of somnolence. Furthermore, there was a lack of comprehensive safety data for tiagabine and levetiracetam, necessitating further research in this area. Larger sample studies were still needed to strengthen the support for these findings and to gain a better understanding of the safety profiles of all the ASMs involved.

## Data Availability

The original contributions presented in the study are included in the article/Supplementary Material, further inquiries can be directed to the corresponding authors.
