# Supplementary material for: Effectiveness and safety of single anti-seizure medication as adjunctive therapy for drug-resistant focal epilepsy based on network meta-analysis
Source: Front Pharmacol. 2025 Apr 25;16:1500475. doi: 10.3389/fphar.2025.1500475 (PMC12061871; doi:10.3389/fphar.2025.1500475)

**Supplementary Material**

**Supplementary Method 1** Search strategy

**Supplementary Table 1** Risk of bias for randomized controlled trials

**Supplementary Table 2** Results of network and traditional paired meta-analysis for dizziness

**Supplementary Table 3** Results of network and traditional paired meta-analysis for somnolence

**Supplementary Table 4** Results of network and traditional paired meta-analysis for headache

**Supplementary Table 5** Results of network and traditional paired meta-analysis for ataxia

**Supplementary Table 6** Results of network and traditional paired meta-analysis for diplopia

**Supplementary Table 7** Results of network and traditional paired meta-analysis for fatigue

**Supplementary Table 8** Results of network and traditional paired meta-analysis for nausea

**Supplementary Table 9** Result of test of inconsistency based on the loop inconsistency method for 50% response rate

**Supplementary Table 10** Result of test of inconsistency based on the loop inconsistency method for dizziness

**Supplementary Table 11** Result of test of inconsistency based on the loop inconsistency method for somnolence

**Supplementary Table 12** Result of test of inconsistency based on the loop inconsistency method for headache

**Supplementary Table 13** Result of test of inconsistency based on the loop inconsistency method for nausea

**Supplementary Figure 1** Network plot for dizziness

**Supplementary Figure 2** Network plot for somnolence

**Supplementary Figure 3** Network plot for headache

**Supplementary Figure 4** Network plot for ataxia

**Supplementary Figure 5** Network plot for diplopia

**Supplementary Figure 6** Network plot for fatigue

**Supplementary Figure 7** Network plot for nausea

**Supplementary Figure 8** Funnel plot for 50% response rate

**Supplementary Figure 9** Funnel plot for dizziness

**Supplementary Figure 10** Funnel plot for somnolence

**Supplementary Figure 11** Funnel plot for headache

**Supplementary Figure 12** Funnel plot for ataxia

**Supplementary Figure 13** Funnel plot for diplopia

**Supplementary Figure 14** Funnel plot for fatigue

**Supplementary Figure 15** Funnel plot for nausea

**Supplementary Method 1** Search strategy

1. **PubMed**

((drug-resistant) OR (medication-resistant) OR (intractable) OR (refractory) OR (uncontrolled) OR (drug refractory) OR (pharmacoresistant) OR (complex) OR (partial) OR (partial-onset) OR (focal)) AND (epilepsy OR seizure), Filters applied: Randomized Controlled Trial /(1261)

1. **Embase**

(‘drug-resistant’ OR ‘medication resistant’ OR intractable OR refractory OR uncontrolled OR ‘drug refractory’ OR ((‘drug’/exp OR drug) AND refractory) OR pharmacoresistant OR ‘complex’/exp OR complex OR partial OR ‘partial-onset’ OR focal) AND (‘epilepsy’/exp OR epilepsy OR ‘seizure’/exp OR seizure) AND [randomized controlled trial]/lim AND [embase]/lim /2040

1. **Cochrane Library**
2. (drug-resistant):ti,ab,kw
3. (medication-resistant):ti,ab,kw
4. (intractable):ti,ab,kw
5. (refractory):ti,ab,kw
6. (uncontrolled):ti,ab,kw
7. (drug refractory):ti,ab,kw
8. (pharmacoresistant).ti,ab,kw
9. (complex):ti,ab,kw
10. (partial).ti,ab,kw
11. (partial-onset):ti,ab,kw
12. (focal):ti,ab,kw
13. #1 OR #2 OR #3 OR #4 OR #5 OR #6 OR #7 OR #8 OR #9 OR #10 OR #11
14. MeSH descriptor: [Epilepsy] explode all trees
15. MeSH descriptor: [Seizures] explode all trees
16. #13 OR #14
17. #12 AND #15 /(2075)

**Supplementary Table 1** Risk of bias for randomized controlled trials

| **Study** | **Year** | **Randomisation** | **Effect of Assignment to Intervention** | **Missing Outcome Data** | **Measurement of the Outcome** | **Selection of Reported Result** | **Overall Risk** |
| --- | --- | --- | --- | --- | --- | --- | --- |
| Anhut | 1994 | Low risk | Low risk | Low risk | Low risk | Low risk | Low risk |
| Arroyo | 2004 | Low risk | Low risk | High risk | Low risk | Low risk | Low risk |
| Barcs | 2000 | Low risk | Low risk | Low risk | Low risk | Low risk | Low risk |
| Baulac | 2010 | Low risk | Low risk | Low risk | Low risk | Low risk | Low risk |
| Ben-Menachem | 1996 | Low risk | Low risk | Some concerns | Low risk | Low risk | Some concerns |
| Ben-Menachem | 1997 | Low risk | Low risk | Low risk | Some concerns | Low risk | Some concerns |
| Ben-Menachem | 2010 | Low risk | Low risk | Some concerns | Low risk | Some concerns | High risk |
| Beydoun | 2005 | Low risk | Low risk | Some concerns | Low risk | Some concerns | High risk |
| Biton | 2011 | Low risk | Low risk | Low risk | Low risk | Low risk | Low risk |
| Brodie | 2004 | Low risk | Low risk | Low risk | Some concerns | Low risk | Some concerns |
| Brodie | 2005 | Low risk | Low risk | Low risk | Low risk | Low risk | Low risk |
| Bruni | 2000 | Low risk | Low risk | Some concerns | Low risk | Low risk | Some concerns |
| Cereghino | 2000 | Low risk | Low risk | Low risk | Low risk | Low risk | Low risk |
| Chadwick | 2000 | Low risk | Low risk | Low risk | Low risk | Low risk | Low risk |
| Chung | 2020 | Low risk | Low risk | Low risk | Low risk | Low risk | Low risk |
| Faught | 1997 | Low risk | Low risk | Low risk | Low risk | Some concerns | Some concerns |
| Faught | 1996 | Low risk | Low risk | Low risk | Low risk | Some concerns | Some concerns |
| French | 1996 | Low risk | Low risk | Some concerns | Low risk | Low risk | Some concerns |
| French | 2003 | Low risk | Low risk | Some concerns | Low risk | Low risk | Some concerns |
| French | 2010 | Low risk | Low risk | Low risk | Low risk | Low risk | Low risk |
| French | 2014 | Low risk | Low risk | Low risk | Low risk | Low risk | Low risk |
| French | 2016 | Low risk | Low risk | Some concerns | Low risk | Low risk | Some concerns |
| French | 2021 | Low risk | Low risk | Low risk | Low risk | Low risk | Low risk |
| Gil-Nagel | 2009 | Low risk | Low risk | Some concerns | Low risk | Low risk | Some concerns |
| Guberman | 2002 | Low risk | Low risk | Some concerns | Low risk | Low risk | Some concerns |
| Hogan | 2014 | Low risk | Low risk | Some concerns | Low risk | Some concerns | High risk |
| Hong | 2016 | Low risk | Low risk | Low risk | Some concerns | Low risk | Some concerns |
| Inoue | 2021 | Low risk | Low risk | Low risk | Low risk | Low risk | Low risk |
| Kälviäinen | 1998 | Low risk | Low risk | Low risk | Low risk | Some concerns | Some concerns |
| Klein | 2015 | Low risk | Low risk | Some concerns | Low risk | Low risk | Some concerns |
| Krauss | 2020 | Low risk | Low risk | Low risk | Low risk | Low risk | Low risk |
| Lee | 2009 | Low risk | Low risk | Low risk | Low risk | Some concerns | Some concerns |
| Lindberger | 2000 | Low risk | Low risk | Low risk | Low risk | Low risk | Low risk |
| Matsuo | 1993 | Low risk | Low risk | Low risk | Low risk | Low risk | Low risk |
| Naritoku | 2007 | Low risk | Low risk | Low risk | Low risk | Low risk | Low risk |
| Nishida | 2018 | Low risk | Low risk | Low risk | Low risk | Low risk | Low risk |
| No authors listed | 1993 | Low risk | Low risk | Low risk | Low risk | Some concerns | Some concerns |
| No authors listed | 1990 | Low risk | Low risk | Low risk | Low risk | Low risk | Low risk |
| Peltola | 2009 | Low risk | Low risk | Low risk | Low risk | Low risk | Low risk |
| Privitera | 1996 | Low risk | Low risk | Some concerns | Low risk | Low risk | Some concerns |
| Sackellares | 2004 | Low risk | Low risk | Low risk | Low risk | Low risk | Low risk |
| Schmidt | 1993 | Low risk | Low risk | Low risk | Low risk | Low risk | Low risk |
| Sharief | 1996 | Low risk | Low risk | Low risk | Low risk | Low risk | Low risk |
| Shorvon | 2000 | Low risk | Low risk | Low risk | Low risk | Low risk | Low risk |
| Tassinari | 1996 | Low risk | Low risk | Some concerns | Low risk | Some concerns | High risk |
| Tsai | 2006 | Low risk | Low risk | Low risk | Low risk | Low risk | Low risk |
| Uthman | 1998 | Low risk | Low risk | Low risk | Low risk | Some concerns | Some concerns |
| Wu | 2009 | Low risk | Low risk | Some concerns | Low risk | Low risk | Some concerns |
| Xiao | 2009 | Low risk | Low risk | Low risk | Low risk | Low risk | Low risk |
| Yamauchi | 2006 | Low risk | Low risk | Low risk | Low risk | Low risk | Low risk |
| Yen | 2000 | Low risk | Low risk | Low risk | Low risk | Some concerns | Some concerns |
| Zaccara | 2014 | Low risk | Low risk | Low risk | Low risk | Low risk | Low risk |
| Zhou | 2008 | Low risk | Low risk | Low risk | Low risk | Some concerns | Some concerns |

**Supplementary Table 2** Results of network and traditional paired meta-analysis for dizziness

| **Placebo** | **1.88 (1.09,3.24)** | **1.57 (1.04,2.37)** | **2.81 (1.87,4.20)** | **2.55 (1.80,3.61)** | 1.27 (0.88,1.84) | **1.58 (1.04,2.42)** | **1.78 (1.20,2.63)** | 5.31 (0.60,46.66) | **2.55 (1.67,3.91)** | **5.34 (3.23,8.83)** | **2.88 (2.21,3.77)** | **2.50 (1.02,6.15)** | **3.20 (1.44,7.13)** | **2.02 (1.32,3.10)** | **1.67 (1.28,2.17)** | 1.70 (0.61,4.73) | **3.35 (1.71,6.55)** |
| --- | --- | --- | --- | --- | --- | --- | --- | --- | --- | --- | --- | --- | --- | --- | --- | --- | --- |
| 1.71 (0.89,3.31) | **Brivaracetam** | 0.83 (0.42,1.65) | 1.49 (0.76,2.94) | 1.35 (0.71,2.58) | 0.68 (0.35,1.31) | 0.84 (0.42,1.68) | 0.95 (0.48,1.85) | 2.82 (0.30,26.51) | 1.36 (0.68,2.71) | **2.84 (1.35,5.95)** | 1.53 (0.84,2.81) | 1.33 (0.47,3.80) | 1.70 (0.65,4.48) | 1.07 (0.54,2.15) | 0.89 (0.48,1.63) | 0.90 (0.28,2.88) | 1.78 (0.75,4.22) |
| **1.59 (1.18,2.14)** | / | **Cenobamate** | **1.79 (1.00,3.18)** | 1.62 (0.94,2.78) | 0.81 (0.47,1.41) | 1.01 (0.56,1.82) | 1.13 (0.64,2.00) | 3.38 (0.37,30.86) | 1.63 (0.90,2.94) | **3.40 (1.77,6.51)** | **1.84 (1.12,3.00)** | 1.59 (0.59,4.28) | 2.04 (0.83,5.01) | 1.29 (0.71,2.33) | 1.06 (0.65,1.73) | 1.08 (0.36,3.26) | 2.13 (0.97,4.68) |
| **2.85 (2.10,3.87)** | / | / | **Eslicarbazepine acetate** | 0.91 (0.53,1.55) | **0.45 (0.26,0.78)** | 0.56 (0.31,1.01) | 0.63 (0.36,1.11) | 1.89 (0.21,17.26) | 0.91 (0.51,1.64) | **1.90 (1.00,3.63)** | 1.03 (0.63,1.67) | 0.89 (0.33,2.39) | 1.14 (0.47,2.80) | 0.72 (0.40,1.30) | **0.59 (0.37,0.96)** | 0.60 (0.20,1.82) | 1.19 (0.55,2.61) |
| **2.54 (1.86,3.47)** | / | / | / | **Gabapentin** | **0.50 (0.30,0.83)** | 0.62 (0.36,1.08) | 0.70 (0.42,1.17) | 2.09 (0.23,18.84) | 1.00 (0.58,1.74) | **2.10 (1.14,3.87)** | 1.13 (0.75,1.71) | 0.98 (0.38,2.58) | 1.26 (0.53,3.01) | 0.79 (0.46,1.38) | 0.66 (0.42,1.02) | 0.67 (0.23,1.97) | 1.32 (0.62,2.80) |
| 1.35 (0.63,2.91) | / | / | / | / | **Lacosamide** | 1.24 (0.71,2.18) | 1.40 (0.82,2.39) | 4.17 (0.46,37.80) | **2.01 (1.14,3.53)** | **4.19 (2.25,7.83)** | **2.27 (1.44,3.57)** | 1.97 (0.75,5.19) | **2.52 (1.04,6.07)** | 1.59 (0.90,2.79) | 1.31 (0.84,2.06) | 1.33 (0.45,3.96) | **2.63 (1.22,5.66)** |
| 1.59 (0.97,2.63) | / | / | / | / | / | **Lamotrigine** | 1.12 (0.63,2.00) | 3.35 (0.37,30.70) | 1.61 (0.88,2.94) | **3.37 (1.75,6.51)** | **1.82 (1.10,3.01)** | 1.58 (0.59,4.27) | 2.02 (0.82,5.00) | 1.28 (0.70,2.33) | 1.05 (0.64,1.74) | 1.07 (0.35,3.25) | 2.11 (0.96,4.68) |
| **1.71 (1.05,2.78)** | / | / | / | / | / | / | **Levetiracetam** | 2.98 (0.33,27.13) | 1.43 (0.80,2.56) | **3.00 (1.59,5.67)** | **1.62 (1.06,2.48)** | 1.41 (0.53,3.74) | 1.80 (0.74,4.38) | 1.13 (0.64,2.03) | 0.94 (0.59,1.50) | 0.95 (0.32,2.85) | 1.88 (0.87,4.09) |
| 5.31 (0.66,43.04) | / | / | / | / | / | / | / | **Natalizumab** | 0.48 (0.05,4.40) | 1.01 (0.11,9.35) | 0.54 (0.06,4.85) | 0.47 (0.04,4.95) | 0.60 (0.06,6.11) | 0.38 (0.04,3.48) | 0.31 (0.04,2.80) | 0.32 (0.03,3.53) | 0.63 (0.06,6.13) |
| **2.57 (1.90,3.48)** | / | / | / | / | / | / | / | / | **Oxcarbazepine** | **2.09 (1.08,4.04)** | 1.13 (0.68,1.87) | 0.98 (0.36,2.65) | 1.25 (0.51,3.10) | 0.79 (0.43,1.45) | 0.65 (0.40,1.08) | 0.66 (0.22,2.02) | 1.31 (0.59,2.90) |
| **5.37 (3.70,7.78)** | / | / | / | / | / | / | / | / | / | **Perampanel** | **0.54 (0.31,0.96)** | 0.47 (0.17,1.31) | 0.60 (0.23,1.54) | **0.38 (0.20,0.73)** | **0.31 (0.18,0.55)** | **0.32 (0.10,1.00)** | 0.63 (0.27,1.45) |
| **2.96 (2.30,3.81)** | / | / | / | 1.10 (0.62,1.96) | / | / | 1.44 (0.996,2.44) | / | / | / | **Pregabalin** | 0.87 (0.34,2.22) | 1.11 (0.48,2.58) | 0.70 (0.42,1.16) | **0.58 (0.40,0.84)** | 0.59 (0.20,1.70) | 1.16 (0.56,2.39) |
| **2.51 (1.13,5.56)** | / | / | / | / | / | / | / | / | / | / | / | **Remacemid** | 1.28 (0.38,4.26) | 0.81 (0.30,2.18) | 0.67 (0.26,1.70) | 0.68 (0.17,2.65) | 1.34 (0.44,4.10) |
| **3.21 (1.86,5.52)** | / | / | / | / | / | / | / | / | / | / | / | / | **Rufinamide** | 0.63 (0.25,1.56) | 0.52 (0.22,1.21) | 0.53 (0.14,1.94) | 1.05 (0.37,2.97) |
| **2.00 (1.47,2.72)** | / | / | / | / | / | / | / | / | / | / | / | / | / | **Tiagabine** | 0.83 (0.50,1.36) | 0.84 (0.28,2.55) | 1.66 (0.75,3.67) |
| **1.65 (1.30,2.09)** | / | / | / | / | / | / | / | / | / | / | / | / | / | / | **Topiramate** | 1.02 (0.35,2.93) | 2.01 (0.98,4.12) |
| 1.70 (0.73,3.93) | / | / | / | / | / | / | / | / | / | / | / | / | / | / | / | **Vigabatrin** | 1.97 (0.58,6.72) |
| **3.41 (1.85,6.29)** | / | / | / | / | / | / | / | / | / | / | / | / | / | / | / | / | **Zonisamide** |

**Note:** Comparisons between anti-seizure medications should be read from right to left, and the results are all comparisons between treatments defined on the bottom right and treatments defined on the top left. The table is divided into lower left and upper right sections with anti-seizure medications as the dividing line. The upper right represents the network comparison results, and the lower left part represents the direct comparison results. For comparison results, when relative risk (RR) <1, tended to define treatment on the left, when RR >1, treatment tends to be defined to the right. Significant results are in bold and underline, and "/" means that the results are not available.

**Supplementary Table 3** Results of network and traditional paired meta-analysis for somnolence

| **Placebo** | **1.93 (1.32,2.84)** | **2.59 (1.79,3.73)** | 1.15 (0.83,1.60) | **1.97 (1.55,2.50)** | 1.15 (0.76,1.73) | 1.49 (0.93,2.40) | **1.63 (1.27,2.09)** | 0.15 (0.01,2.86) | **2.26 (1.64,3.11)** | 1.31 (0.95,1.82) | **1.91 (1.55,2.36)** | 0.78 (0.31,1.94) | 1.73 (0.85,3.50) | 0.92 (0.41,2.04) | **2.27 (1.80,2.85)** | **2.60 (1.46,4.61)** |
| --- | --- | --- | --- | --- | --- | --- | --- | --- | --- | --- | --- | --- | --- | --- | --- | --- |
| 1.61 (0.92,2.81) | **Brivaracetam** | 1.34 (0.79,2.28) | **0.60 (0.36,0.99)** | 1.02 (0.66,1.59) | 0.59 (0.34,1.05) | 0.77 (0.42,1.43) | 0.84 (0.53,1.33) | 0.08 (0.00,1.52) | 1.17 (0.71,1.92) | 0.68 (0.41,1.12) | 0.99 (0.64,1.54) | 0.40 (0.15,1.08) | 0.90 (0.40,2.00) | 0.47 (0.20,1.15) | 1.17 (0.76,1.83) | 1.34 (0.67,2.69) |
| **2.59 (1.77,3.80)** | / | **Cenobamate** | **0.44 (0.27,0.73)** | 0.76 (0.49,1.18) | **0.44 (0.26,0.77)** | 0.58 (0.32,1.05) | **0.63 (0.40,0.98)** | 0.06 (0.00,1.13) | 0.87 (0.54,1.42) | **0.51 (0.31,0.83)** | 0.74 (0.49,1.13) | **0.30 (0.11,0.80)** | 0.67 (0.30,1.48) | **0.35 (0.15,0.85)** | 0.88 (0.57,1.35) | 1.00 (0.51,1.98) |
| 1.15 (0.85,1.55) | / | / | **Eslicarbazepine acetate** | **1.71 (1.14,2.57)** | 1.00 (0.59,1.69) | 1.30 (0.73,2.31) | 1.42 (0.94,2.14) | 0.13 (0.01,2.53) | **1.97 (1.24,3.11)** | 1.14 (0.72,1.81) | **1.67 (1.13,2.46)** | 0.68 (0.26,1.79) | 1.51 (0.69,3.28) | 0.80 (0.34,1.89) | **1.97 (1.32,2.95)** | **2.26 (1.17,4.38)** |
| **1.96 (1.47,2.60)** | / | / | / | **Gabapentin** | **0.58 (0.36,0.94)** | 0.76 (0.44,1.29) | 0.83 (0.59,1.15) | 0.08 (0.00,1.46) | 1.15 (0.77,1.70) | **0.67 (0.44,1.00)** | 0.97 (0.73,1.30) | 0.40 (0.15,1.01) | 0.88 (0.42,1.85) | 0.47 (0.20,1.07) | 1.15 (0.83,1.60) | 1.32 (0.71,2.45) |
| 1.24 (0.54,2.86) | / | / | / | / | **Lacosamide** | 1.30 (0.70,2.43) | 1.42 (0.88,2.30) | 0.13 (0.01,2.56) | **1.97 (1.17,3.31)** | 1.14 (0.68,1.93) | **1.67 (1.05,2.64)** | 0.68 (0.25,1.85) | 1.51 (0.67,3.41) | 0.80 (0.33,1.96) | **1.98 (1.23,3.17)** | **2.26 (1.12,4.58)** |
| 1.51 (0.91,2.51) | / | / | / | / | / | **Lamotrigine** | 1.09 (0.64,1.87) | 0.10 (0.01,1.99) | 1.51 (0.85,2.68) | 0.88 (0.49,1.56) | 1.28 (0.76,2.15) | 0.52 (0.19,1.46) | 1.16 (0.50,2.71) | 0.61 (0.24,1.56) | 1.52 (0.90,2.58) | 1.74 (0.83,3.66) |
| **1.55 (1.13,2.14)** | / | / | / | / | / | / | **Levetiracetam** | 0.09 (0.00,1.77) | 1.39 (0.92,2.08) | 0.81 (0.53,1.22) | 1.18 (0.90,1.54) | 0.48 (0.19,1.23) | 1.06 (0.50,2.25) | 0.56 (0.24,1.30) | 1.39 (0.99,1.96) | 1.60 (0.85,2.99) |
| 0.15 (0.01,2.82) | / | / | / | / | / | / | / | **Natalizumab** | 14.91 (0.78,286.08) | 8.65 (0.45,166.13) | 12.63 (0.66,239.96) | 5.14 (0.24,111.25) | 11.42 (0.56,234.16) | 6.05 (0.29,126.97) | 14.97 (0.79,284.87) | 17.14 (0.86,341.76) |
| **2.27 (1.72,3.00)** | / | / | / | / | / | / | / | / | **Oxcarbazepine** | **0.58 (0.37,0.92)** | 0.85 (0.58,1.24) | **0.34 (0.13,0.90)** | 0.77 (0.35,1.66) | **0.41 (0.17,0.96)** | 1.00 (0.68,1.49) | 1.15 (0.60,2.22) |
| 1.31 (0.98,1.75) | / | / | / | / | / | / | / | / | / | **Perampanel** | 1.46 (0.99,2.15) | 0.59 (0.23,1.56) | 1.32 (0.61,2.87) | 0.70 (0.29,1.66) | **1.73 (1.16,2.58)** | **1.98 (1.02,3.84)** |
| **1.97 (1.50,2.58)** | / | / | / | 1.00 (0.64,1.55) | / | / | 1.09 (0.83,1.42) | / | / | / | **Pregabalin** | 0.41 (0.16,1.04) | 0.90 (0.43,1.89) | 0.48 (0.21,1.10) | 1.19 (0.87,1.62) | 1.36 (0.74,2.50) |
| 0.62 (0.14,2.77) | / | / | / | / | / | / | / | / | / | / | / | **Remacemid** | 2.22 (0.70,7.04) | 1.18 (0.35,3.96) | **2.91 (1.14,7.45)** | **3.34 (1.14,9.80)** |
| 1.73 (0.90,3.33) | / | / | / | / | / | / | / | / | / | / | / | / | **Rufinamide** | 0.53 (0.18,1.54) | 1.31 (0.62,2.75) | 1.50 (0.60,3.73) |
| 0.92 (0.43,1.95) | / | / | / | / | / | / | / | / | / | / | / | / | / | **Tiagabine** | **2.47 (1.08,5.69)** | **2.83 (1.06,7.59)** |
| **2.26 (1.78,2.87)** | / | / | / | / | / | / | / | / | / | / | / | / | / | / | **Topiramate** | 1.15 (0.62,2.12) |
| **2.59 (1.48,4.54)** | / | / | / | / | / | / | / | / | / | / | / | / | / | / | / | **Zonisamide** |

**Note:** Comparisons between anti-seizure medications should be read from right to left, and the results are all comparisons between treatments defined on the bottom right and treatments defined on the top left. The table is divided into lower left and upper right sections with anti-seizure medications as the dividing line. The upper right represents the network comparison results, and the lower left part represents the direct comparison results. For comparison results, when relative risk (RR) <1, tended to define treatment on the left, when RR >1, treatment tends to be defined to the right. Significant results are in bold and underline, and "/" means that the results are not available.

**Supplementary Table 4** Results of network and traditional paired meta-analysis for headache

| **Placebo** | 0.83 (0.57,1.22) | 1.47 (0.96,2.26) | 1.30 (0.90,1.89) | 1.02 (0.73,1.42) | 1.23 (0.92,1.65) | 1.18 (0.92,1.53) | 0.92 (0.71,1.19) | 1.27 (0.43,3.77) | 1.16 (0.94,1.43) | 0.89 (0.58,1.39) | **0.69 (0.54,0.88)** | 1.00 (0.59,1.71) | 1.29 (0.78,2.14) | 1.15 (0.59,2.26) | 0.92 (0.82,1.05) | 1.45 (0.78,2.69) | 0.66 (0.39,1.12) |
| --- | --- | --- | --- | --- | --- | --- | --- | --- | --- | --- | --- | --- | --- | --- | --- | --- | --- |
| 0.83 (0.57,1.22) | **Brivaracetam** | 1.77 (0.99,3.14) | 1.56 (0.92,2.67) | 1.23 (0.74,2.03) | 1.48 (0.91,2.40) | 1.42 (0.90,2.25) | 1.10 (0.70,1.75) | 1.53 (0.49,4.84) | 1.39 (0.90,2.15) | 1.07 (0.60,1.93) | 0.83 (0.53,1.31) | 1.21 (0.63,2.33) | 1.55 (0.82,2.92) | 1.39 (0.64,3.01) | 1.11 (0.74,1.66) | 1.74 (0.84,3.60) | 0.79 (0.41,1.52) |
| 1.47 (0.96,2.26) | / | **Cenobamate** | 0.89 (0.50,1.56) | 0.69 (0.40,1.19) | 0.84 (0.50,1.41) | 0.81 (0.49,1.33) | 0.62 (0.38,1.03) | 0.87 (0.27,2.78) | 0.79 (0.49,1.27) | 0.61 (0.33,1.12) | **0.47 (0.29,0.77)** | 0.68 (0.34,1.35) | 0.88 (0.45,1.70) | 0.78 (0.35,1.74) | **0.63 (0.40,0.98)** | 0.98 (0.46,2.09) | **0.45 (0.23,0.88)** |
| 1.26 (0.79,2.00) | / | / | **Eslicarbazepine acetate** | 0.78 (0.48,1.29) | 0.94 (0.59,1.52) | 0.91 (0.58,1.43) | 0.71 (0.45,1.11) | 0.98 (0.31,3.08) | 0.89 (0.58,1.36) | 0.69 (0.39,1.22) | **0.53 (0.34,0.83)** | 0.77 (0.40,1.48) | 0.99 (0.53,1.86) | 0.89 (0.41,1.91) | 0.71 (0.48,1.05) | 1.11 (0.54,2.29) | **0.50 (0.26,0.97)** |
| 1.08 (0.71,1.64) | / | / | / | **Gabapentin** | 1.21 (0.78,1.88) | 1.16 (0.77,1.76) | 0.90 (0.60,1.35) | 1.25 (0.40,3.88) | 1.14 (0.77,1.68) | 0.88 (0.51,1.52) | **0.68 (0.47,0.98)** | 0.98 (0.53,1.84) | 1.26 (0.69,2.31) | 1.13 (0.54,2.39) | 0.91 (0.64,1.29) | 1.42 (0.70,2.86) | 0.64 (0.35,1.20) |
| 1.23 (0.91,1.66) | / | / | / | / | **Lacosamide** | 0.96 (0.65,1.42) | 0.75 (0.51,1.11) | 1.04 (0.34,3.19) | 0.94 (0.66,1.35) | 0.73 (0.43,1.23) | **0.56 (0.38,0.83)** | 0.82 (0.44,1.50) | 1.05 (0.58,1.88) | 0.94 (0.45,1.95) | 0.75 (0.55,1.03) | 1.18 (0.59,2.34) | **0.53 (0.29,0.98)** |
| 1.18 (0.92,1.53) | / | / | / | / | / | **Lamotrigine** | 0.78 (0.54,1.11) | 1.08 (0.35,3.28) | 0.98 (0.70,1.36) | 0.75 (0.45,1.26) | **0.59 (0.41,0.83)** | 0.85 (0.47,1.53) | 1.09 (0.62,1.92) | 0.97 (0.47,2.00) | 0.78 (0.59,1.03) | 1.22 (0.63,2.39) | **0.56 (0.31,1.00)** |
| 1.07 (0.75,1.53) | / | / | / | / | / | / | **Levetiracetam** | 1.39 (0.46,4.23) | 1.26 (0.90,1.76) | 0.97 (0.58,1.62) | 0.75 (0.55,1.03) | 1.09 (0.60,1.97) | 1.40 (0.80,2.48) | 1.26 (0.61,2.58) | 1.00 (0.75,1.34) | 1.57 (0.80,3.08) | 0.72 (0.40,1.29) |
| 1.28 (0.43,3.77) | / | / | / | / | / | / | / | **Natalizumab** | 0.91 (0.30,2.74) | 0.70 (0.22,2.26) | 0.54 (0.18,1.65) | 0.79 (0.23,2.63) | 1.01 (0.31,3.35) | 0.90 (0.25,3.24) | 0.72 (0.24,2.16) | 1.13 (0.33,3.96) | 0.52 (0.15,1.72) |
| 1.16 (0.94,1.43) | / | / | / | / | / | / | / | / | **Oxcarbazepine** | 0.77 (0.47,1.26) | **0.60 (0.43,0.82)** | 0.87 (0.49,1.54) | 1.11 (0.64,1.92) | 1.00 (0.49,2.01) | 0.80 (0.63,1.02) | 1.25 (0.65,2.40) | **0.57 (0.32,1.00)** |
| 0.89 (0.57,1.39) | / | / | / | / | / | / | / | / | / | **Perampanel** | 0.78 (0.47,1.28) | 1.12 (0.56,2.24) | 1.44 (0.74,2.82) | 1.29 (0.58,2.88) | 1.03 (0.65,1.63) | 1.62 (0.76,3.46) | 0.74 (0.37,1.47) |
| **0.56 (0.42,0.75)** | / | / | / | 0.85 (0.46,1.58) | / | / | 1.26 (0.76,2.09) | / | / | / | **Pregabalin** | 1.45 (0.80,2.60) | **1.86 (1.06,3.26)** | 1.66 (0.81,3.40) | **1.33 (1.01,1.75)** | **2.09 (1.07,4.06)** | 0.95 (0.53,1.70) |
| 1.003 (0.59,1.71) | / | / | / | / | / | / | / | / | / | / | / | **Remacemid** | 1.29 (0.62,2.68) | 1.15 (0.49,2.71) | 0.92 (0.53,1.59) | 1.44 (0.64,3.27) | 0.66 (0.31,1.39) |
| 1.29 (0.78,2.14) | / | / | / | / | / | / | / | / | / | / | / | / | **Rufinamide** | 0.89 (0.39,2.08) | 0.72 (0.43,1.21) | 1.12 (0.50,2.50) | 0.51 (0.24,1.06) |
| 1.15 (0.59,2.26) | / | / | / | / | / | / | / | / | / | / | / | / | / | **Tiagabine** | 0.80 (0.40,1.59) | 1.25 (0.50,3.13) | 0.57 (0.24,1.34) |
| 0.91 (0.78,1.06) | / | / | / | / | / | / | / | / | / | / | / | / | / | / | **Topiramate** | 1.57 (0.83,2.95) | 0.71 (0.41,1.23) |
| 1.45 (0.78,2.69) | / | / | / | / | / | / | / | / | / | / | / | / | / | / | / | **Vigabatrin** | 0.45 (0.20,1.03) |
| 0.65 (0.36,1.18) | / | / | / | / | / | / | / | / | / | / | / | / | / | / | / | / | **Zonisamide** |

**Note:** Comparisons between anti-seizure medications should be read from right to left, and the results are all comparisons between treatments defined on the bottom right and treatments defined on the top left. The table is divided into lower left and upper right sections with anti-seizure medications as the dividing line. The upper right represents the network comparison results, and the lower left part represents the direct comparison results. For comparison results, when relative risk (RR) <1, tended to define treatment on the left, when RR >1, treatment tends to be defined to the right. Significant results are in bold and underline, and "/" means that the results are not available.

**Supplementary Table 5** Results of network and traditional paired meta-analysis for ataxia

| **Placebo** | **3.98 (1.10,14.39)** | **1.82 (1.16,2.83)** | **2.14 (1.14,4.05)** | **3.55 (2.24,5.62)** | **3.56 (2.12,5.99)** | 5.36 (0.66,43.62) | **2.54 (1.67,3.87)** | 8.24 (0.45,152.24) | **18.49 (1.08,317.73)** |
| --- | --- | --- | --- | --- | --- | --- | --- | --- | --- |
| **3.98 (1.12,14.21)** | **Cenobamate** | 0.46 (0.12,1.78) | 0.54 (0.13,2.26) | 0.89 (0.23,3.49) | 0.90 (0.22,3.58) | 1.35 (0.12,15.76) | 0.64 (0.16,2.47) | 2.07 (0.09,50.15) | 4.65 (0.20,105.34) |
| **1.82 (1.19,2.80)** | / | **Gabapentin** | 1.18 (0.54,2.56) | **1.96 (1.03,3.70)** | 1.96 (0.99,3.89) | 2.95 (0.35,25.18) | 1.40 (0.76,2.58) | 4.54 (0.24,86.73) | 10.18 (0.57,181.16) |
| 2.22 (0.88,5.57) | / | / | **Lamotrigine** | 1.66 (0.76,3.62) | 1.66 (0.73,3.77) | 2.50 (0.28,22.34) | 1.18 (0.55,2.54) | 3.84 (0.19,75.99) | 8.62 (0.47,158.87) |
| **3.45 (1.74,6.83)** | / | / | / | **Oxcarbazepine** | 1.00 (0.50,2.01) | 1.51 (0.18,12.92) | 0.72 (0.38,1.33) | 2.32 (0.12,44.46) | 5.21 (0.29,92.87) |
| **3.56 (2.17,5.86)** | / | / | / | / | **Pregabalin** | 1.50 (0.17,13.04) | 0.71 (0.36,1.39) | 2.31 (0.12,44.73) | 5.19 (0.29,93.47) |
| 5.36 (0.67,43.07) | / | / | / | / | / | **Remacemid** | 0.47 (0.06,4.03) | 1.54 (0.04,55.85) | 3.45 (0.10,118.20) |
| **2.55 (1.75,3.71)** | / | / | / | / | / | / | **Topiramate** | 3.24 (0.17,61.80) | 7.28 (0.41,129.08) |
| 8.24 (0.45,149.46) | / | / | / | / | / | / | / | **Vigabatrin** | 2.24 (0.04,131.93) |
| **18.49 (1.10,311.78)** | / | / | / | / | / | / | / | / | **Zonisamide** |

**Note:** Comparisons between anti-seizure medications should be read from right to left, and the results are all comparisons between treatments defined on the bottom right and treatments defined on the top left. The table is divided into lower left and upper right sections with anti-seizure medications as the dividing line. The upper right represents the network comparison results, and the lower left part represents the direct comparison results. For comparison results, when relative risk (RR) <1, tended to define treatment on the left, when RR >1, treatment tends to be defined to the right. Significant results are in bold and underline, and "/" means that the results are not available.

**Supplementary Table 6** Results of network and traditional paired meta-analysis for diplopia

| **Placebo** | **5.71 (2.39,13.65)** | **2.65 (1.42,4.94)** | **1.87 (1.06,3.29)** | **4.68 (2.63,8.32)** | **5.67 (3.61,8.92)** | **2.40 (1.34,4.30)** | 2.15 (0.72,6.43) | 1.00 (0.29,3.43) | **1.86 (1.35,2.57)** | 2.74 (0.56,13.34) |
| --- | --- | --- | --- | --- | --- | --- | --- | --- | --- | --- |
| **5.71 (2.43,13.44)** | **Cenobamate** | 0.46 (0.16,1.35) | **0.33 (0.12,0.92)** | 0.82 (0.29,2.33) | 0.99 (0.37,2.65) | 0.42 (0.15,1.20) | 0.38 (0.09,1.53) | **0.18 (0.04,0.79)** | **0.33 (0.13,0.83)** | 0.48 (0.08,2.92) |
| **2.66 (1.45,4.86)** | / | **Eslicarbazepine acetate** | 0.70 (0.30,1.64) | 1.77 (0.76,4.13) | 2.14 (0.99,4.62) | 0.91 (0.39,2.13) | 0.81 (0.23,2.86) | 0.38 (0.09,1.50) | 0.70 (0.35,1.42) | 1.04 (0.19,5.67) |
| **1.87 (1.07,3.26)** | / | / | **Gabapentin** | **2.51 (1.12,5.64)** | **3.04 (1.47,6.28)** | 1.29 (0.57,2.91) | 1.15 (0.34,3.96) | 0.54 (0.14,2.08) | 1.00 (0.52,1.92) | 1.47 (0.27,7.90) |
| **4.67 (2.70,8.06)** | / | / | / | **Lamotrigine** | 1.21 (0.58,2.52) | 0.51 (0.23,1.16) | 0.46 (0.13,1.58) | **0.21 (0.05,0.83)** | **0.40 (0.21,0.77)** | 0.59 (0.11,3.15) |
| **5.92 (3.06,10.23)** | / | / | / | / | **Oxcarbazepine** | **0.42 (0.20,0.89)** | 0.38 (0.12,1.24) | **0.18 (0.05,0.65)** | **0.33 (0.19,0.57)** | 0.48 (0.09,2.51) |
| **2.40 (1.37,4.22)** | / | / | / | / | / | **Pregabalin** | 0.90 (0.26,3.09) | 0.42 (0.11,1.63) | 0.77 (0.40,1.51) | 1.14 (0.21,6.16) |
| 2.15 (0.74,6.31) | / | / | / | / | / | / | **Remacemid** | 0.46 (0.09,2.41) | 0.86 (0.28,2.70) | 1.27 (0.19,8.71) |
| 1.00 (0.30,3.32) | / | / | / | / | / | / | / | **Tiagabine** | 1.86 (0.52,6.65) | 2.74 (0.37,20.36) |
| **1.91 (1.27,2.86)** | / | / | / | / | / | / | / | / | **Topiramate** | 1.47 (0.29,7.41) |
| 2.74 (0.58,13.00) | / | / | / | / | / | / | / | / | / | **Vigabatrin** |

**Note:** Comparisons between anti-seizure medications should be read from right to left, and the results are all comparisons between treatments defined on the bottom right and treatments defined on the top left. The table is divided into lower left and upper right sections with anti-seizure medications as the dividing line. The upper right represents the network comparison results, and the lower left part represents the direct comparison results. For comparison results, when relative risk (RR) <1, tended to define treatment on the left, when RR >1, treatment tends to be defined to the right. Significant results are in bold and underline, and "/" means that the results are not available.

**Supplementary Table 7** Results of network and traditional paired meta-analysis for fatigue

| **Placebo** | **2.12 (1.30,3.46)** | **2.02 (1.35,3.02)** | 1.08 (0.54,2.18) | **1.81 (1.14,2.88)** | 0.53 (0.05,5.62) | **2.00 (1.34,2.98)** | 1.25 (0.63,2.48) | 2.60 (0.98,6.88) | 2.14 (0.72,6.39) | **2.03 (1.60,2.57)** | 1.52 (0.71,3.27) | **1.99 (1.13,3.51)** |
| --- | --- | --- | --- | --- | --- | --- | --- | --- | --- | --- | --- | --- |
| **2.14 (1.34,3.40)** | **Brivaracetam** | 0.95 (0.51,1.79) | 0.51 (0.22,1.20) | 0.85 (0.44,1.67) | 0.25 (0.02,2.79) | 0.94 (0.50,1.77) | 0.59 (0.25,1.37) | 1.23 (0.41,3.65) | 1.01 (0.31,3.34) | 0.95 (0.56,1.63) | 0.72 (0.29,1.78) | 0.94 (0.44,1.98) |
| **2.03 (1.31,2.99)** | / | **Cenobamate** | 0.54 (0.24,1.20) | 0.90 (0.49,1.66) | 0.26 (0.02,2.88) | 0.99 (0.56,1.74) | 0.62 (0.28,1.37) | 1.29 (0.45,3.69) | 1.06 (0.33,3.40) | 1.00 (0.63,1.60) | 0.75 (0.32,1.79) | 0.98 (0.49,1.97) |
| 1.09 (0.54,2.16) | / | / | **Eslicarbazepine acetate** | 1.67 (0.72,3.87) | 0.49 (0.04,5.74) | 1.84 (0.82,4.13) | 1.16 (0.43,3.07) | 2.40 (0.72,7.96) | 1.98 (0.54,7.23) | 1.87 (0.89,3.91) | 1.41 (0.50,3.96) | 1.84 (0.75,4.52) |
| **1.81 (1.15,2.85)** | / | / | / | **Gabapentin** | 0.29 (0.03,3.25) | 1.10 (0.60,2.03) | 0.69 (0.30,1.58) | 1.44 (0.49,4.22) | 1.18 (0.36,3.87) | 1.12 (0.66,1.88) | 0.84 (0.34,2.05) | 1.10 (0.53,2.28) |
| 0.53 (0.05,5.58) | / | / | / | / | **Natalizumab** | 3.76 (0.34,41.18) | 2.36 (0.20,27.50) | 4.90 (0.38,62.87) | 4.03 (0.30,54.31) | 3.81 (0.36,40.87) | 2.87 (0.24,34.23) | 3.75 (0.33,42.42) |
| **2.00 (1.37,2.93)** | / | / | / | / | / | **Oxcarbazepine** | 0.63 (0.28,1.38) | 1.30 (0.46,3.72) | 1.07 (0.34,3.43) | 1.01 (0.64,1.61) | 0.76 (0.32,1.80) | 1.00 (0.50,1.99) |
| 1.25 (0.64,2.46) | / | / | / | / | / | / | **Perampanel** | 2.08 (0.63,6.83) | 1.71 (0.47,6.21) | 1.62 (0.78,3.34) | 1.22 (0.44,3.39) | 1.59 (0.65,3.87) |
| 2.59 (0.99,6.80) | / | / | / | / | / | / | / | **Pregabalin** | 0.82 (0.19,3.56) | 0.78 (0.29,2.12) | 0.59 (0.17,2.02) | 0.76 (0.25,2.36) |
| 2.14 (0.73,6.33) | / | / | / | / | / | / | / | / | **Remacemid** | 0.95 (0.31,2.89) | 0.71 (0.19,2.69) | 0.93 (0.27,3.18) |
| **1.97 (1.44,2.69)** | / | / | / | / | / | / | / | / | / | **Topiramate** | 0.75 (0.34,1.67) | 0.98 (0.53,1.82) |
| 1.52 (0.73,3.19) | / | / | / | / | / | / | / | / | / | / | **Vigabatrin** | 1.31 (0.50,3.38) |
| **1.99 (1.15,3.45)** | / | / | / | / | / | / | / | / | / | / | / | **Zonisamide** |

**Note:** Comparisons between anti-seizure medications should be read from right to left, and the results are all comparisons between treatments defined on the bottom right and treatments defined on the top left. The table is divided into lower left and upper right sections with anti-seizure medications as the dividing line. The upper right represents the network comparison results, and the lower left part represents the direct comparison results. For comparison results, when relative risk (RR) <1, tended to define treatment on the left, when RR >1, treatment tends to be defined to the right. Significant results are in bold and underline, and "/" means that the results are not available.

**Supplementary Table 8** Results of network and traditional paired meta-analysis for nausea

| **Placebo** | **3.30 (1.50,7.26)** | **3.34 (1.85,6.00)** | 0.76 (0.37,1.56) | 0.67 (0.31,1.47) | **2.08 (1.24,3.47)** | 1.48 (0.71,3.09) | 3.19 (0.35,29.09) | **2.75 (1.97,3.83)** | 0.99 (0.51,1.92) | 0.56 (0.18,1.72) | 1.13 (0.46,2.76) | 2.37 (0.56,9.97) | 1.25 (0.69,2.27) |
| --- | --- | --- | --- | --- | --- | --- | --- | --- | --- | --- | --- | --- | --- |
| 3.00 (1.14,0.67) | **Cenobamate** | 1.01 (0.38,2.70) | **0.23 (0.08,0.67)** | **0.20 (0.07,0.62)** | 0.63 (0.25,1.61) | 0.45 (0.15,1.32) | 0.97 (0.09,10.11) | 0.83 (0.35,1.96) | **0.30 (0.11,0.84)** | **0.17 (0.04,0.67)** | 0.34 (0.10,1.13) | 0.72 (0.14,3.70) | 0.38 (0.14,1.02) |
| 3.00 (0.17,0.54) | / | **Eslicarbazepine acetate** | **0.23 (0.09,0.58)** | **0.20 (0.08,0.54)** | 0.62 (0.29,1.36) | 0.44 (0.17,1.14) | 0.96 (0.10,9.41) | 0.82 (0.42,1.62) | **0.30 (0.12,0.72)** | **0.17 (0.05,0.59)** | **0.34 (0.12,0.99)** | 0.71 (0.15,3.35) | **0.38 (0.16,0.87)** |
| 1.31 (0.64,2.69) | / | / | **Gabapentin** | 0.88 (0.31,2.55) | **2.72 (1.13,6.58)** | 1.94 (0.69,5.43) | 4.18 (0.41,42.77) | **3.60 (1.63,7.95)** | 1.30 (0.49,3.45) | 0.74 (0.20,2.78) | 1.48 (0.47,4.66) | 3.11 (0.63,15.50) | 1.65 (0.65,4.17) |
| 1.49 (0.68,3.23) | / | / | / | **Lacosamide** | **3.08 (1.21,7.83)** | 2.19 (0.75,6.41) | 4.73 (0.45,49.34) | **4.08 (1.75,9.51)** | 1.47 (0.53,4.08) | 0.83 (0.21,3.25) | 1.67 (0.51,5.48) | 3.52 (0.69,18.03) | 1.86 (0.70,4.95) |
| **0.48 (0.29,0.81)** | / | / | / | / | **Lamotrigine** | 0.71 (0.29,1.75) | 1.54 (0.16,14.87) | 1.32 (0.72,2.44) | 0.48 (0.21,1.10) | **0.27 (0.08,0.93)** | 0.54 (0.19,1.53) | 1.14 (0.25,5.25) | 0.60 (0.28,1.32) |
| 0.88 (0.40,1.94) | / | / | / | / | / | **Levetiracetam** | 2.16 (0.21,22.21) | 1.86 (0.83,4.18) | 0.67 (0.25,1.81) | 0.38 (0.14,1.07) | 0.76 (0.24,2.44) | 1.61 (0.32,8.07) | 0.85 (0.33,2.19) |
| 0.31 (0.03,2.86) | / | / | / | / | / | / | **Natalizumab** | 0.86 (0.09,8.06) | 0.31 (0.03,3.13) | 0.18 (0.01,2.10) | 0.35 (0.03,3.84) | 0.74 (0.05,10.39) | 0.39 (0.04,3.88) |
| **0.37 (0.25,0.53)** | / | / | / | / | / | / | / | **Oxcarbazepine** | **0.36 (0.17,0.76)** | **0.20 (0.06,0.66)** | 0.41 (0.16,1.07) | 0.86 (0.20,3.77) | **0.46 (0.23,0.90)** |
| 1.01 (0.52,1.95) | / | / | / | / | / | / | / | / | **Perampanel** | 0.57 (0.15,2.07) | 1.13 (0.37,3.46) | 2.39 (0.49,11.61) | 1.27 (0.52,3.07) |
| 0.49 (0.09,2.82) | / | / | / | / | / | **4.98 (1.46,16.99)** | / | / | / | **Pregabalin** | 2.01 (0.48,8.42) | 4.23 (0.69,26.09) | 2.24 (0.63,7.93) |
| 0.89 (0.36,2.18) | / | / | / | / | / | / | / | / | / | / | **Tiagabine** | 2.11 (0.39,11.47) | 1.11 (0.38,3.27) |
| 0.42 (0.10,1.77) | / | / | / | / | / | / | / | / | / | / | / | **Topiramate** | 0.53 (0.11,2.50) |
| 0.80 (0.44,1.44) | / | / | / | / | / | / | / | / | / | / | / | / | **Zonisamide** |

**Note:** Comparisons between anti-seizure medications should be read from right to left, and the results are all comparisons between treatments defined on the bottom right and treatments defined on the top left. The table is divided into lower left and upper right sections with anti-seizure medications as the dividing line. The upper right represents the network comparison results, and the lower left part represents the direct comparison results. For comparison results, when relative risk (RR) <1, tended to define treatment on the left, when RR >1, treatment tends to be defined to the right. Significant results are in bold and underline, and "/" means that the results are not available.

**Supplementary Table 9** Result of test of inconsistency based on the loop inconsistency method for 50% response rate

| **Loop** | **IF** | **seIF** | **z_value** | **p_value** | **95%_CI** | **Loop_Heterogeneity_tau^2^** |
| --- | --- | --- | --- | --- | --- | --- |
| Placebo-Gabapentin-Vigabatrin | 0.389 | 0.287 | 1.358 | 0.174 | (0.00,0.95) | 0 |
| Placebo-Levetiracetam-Vigabatrin | 0.243 | 0.515 | 0.472 | 0.637 | (0.00,1.25) | 0.182 |
| Placebo-Gabapentin-Vigabatrin | 0.156 | 0.477 | 0.327 | 0.744 | (0.00,1.09) | 0.157 |

**Supplementary Table 10** Result of test of inconsistency based on the loop inconsistency method for dizziness

| **Loop** | **IF** | **seIF** | **z_value** | **p_value** | **95%_CI** | **Loop_Heterogeneity_tau^2^** |
| --- | --- | --- | --- | --- | --- | --- |
| Placebo-Levetiracetam-Pregabalin | 0.2 | 0.396 | 0.505 | 0.613 | (0.00,0.98) | 0.053 |
| Placebo-Gabapentin-Pregabalin | 0.062 | 0.355 | 0.173 | 0.862 | (0.00,0.76) | 0 |

**Supplementary Table 11** Result of test of inconsistency based on the loop inconsistency method for somnolence

| **Loop** | **IF** | **seIF** | **z_value** | **p_value** | **95%_CI** | **Loop_Heterogeneity_tau^2^** |
| --- | --- | --- | --- | --- | --- | --- |
| Placebo-Levetiracetam-Pregabalin | 0.152 | 0.32 | 0.475 | 0.635 | (0.00,0.78) | 0.036 |
| Placebo-Gabapentin-Pregabalin | 0.006 | 0.373 | 0.015 | 0.988 | (0.00,0.74) | 0.041 |

**Supplementary Table 12** Result of test of inconsistency based on the loop inconsistency method for headache

| **Loop** | **IF** | **seIF** | **z_value** | **p_value** | **95%_CI** | **Loop_Heterogeneity_tau^2^** |
| --- | --- | --- | --- | --- | --- | --- |
| Placebo-Levetiracetam-Pregabalin | 0.886 | 0.332 | 2.667 | 0.008 | (0.23,1.54) | 0 |
| Placebo-Gabapentin-Pregabalin | 0.518 | 0.399 | 1.3 | 0.194 | (0.00,1.30) | 0 |

**Supplementary Table 13** Result of test of inconsistency based on the loop inconsistency method for nausea

| **Loop** | **IF** | **seIF** | **z_value** | **p_value** | **95%_CI** | **Loop_Heterogeneity_tau^2^** |
| --- | --- | --- | --- | --- | --- | --- |
| Placebo-Levetiracetam-Pregabalin | 2.185 | 1.159 | 1.885 | 0.059 | (0.00,4.46) | 0 |

**Supplementary Figure 1** Network plot for dizziness

**
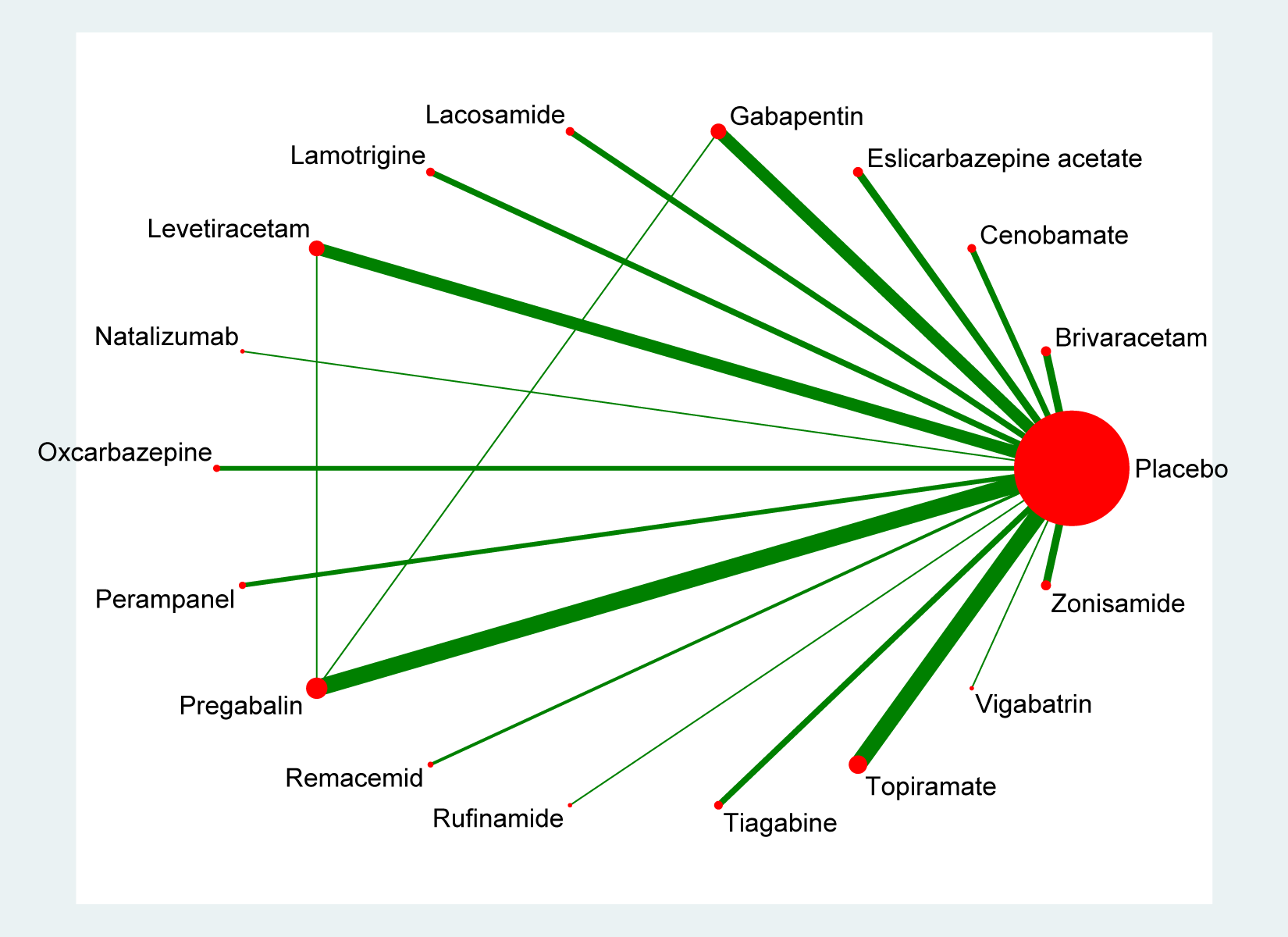
**

**Supplementary Figure 2** Network plot for somnolence

**
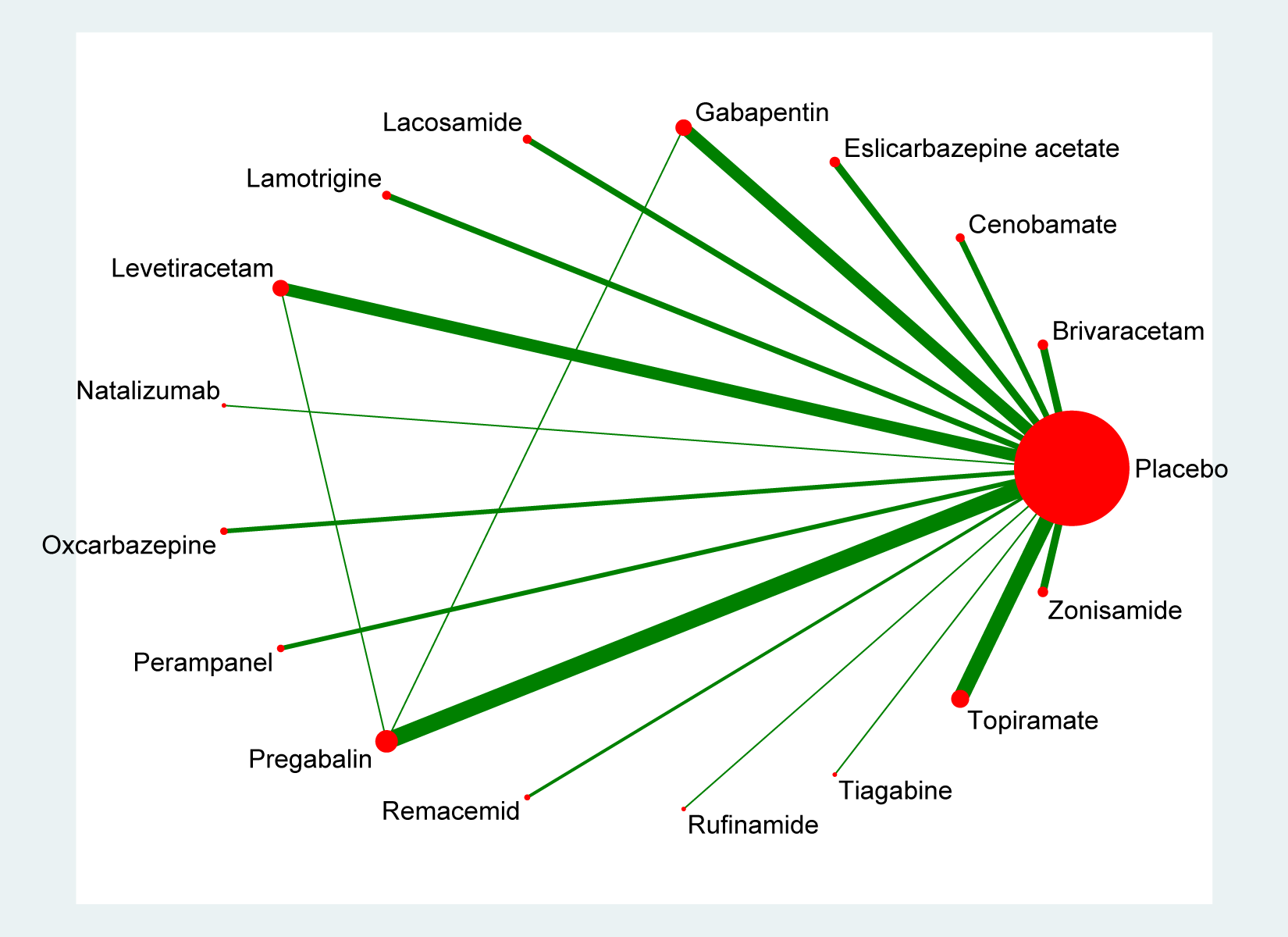
**

**Supplementary Figure 3** Network plot for headache

**
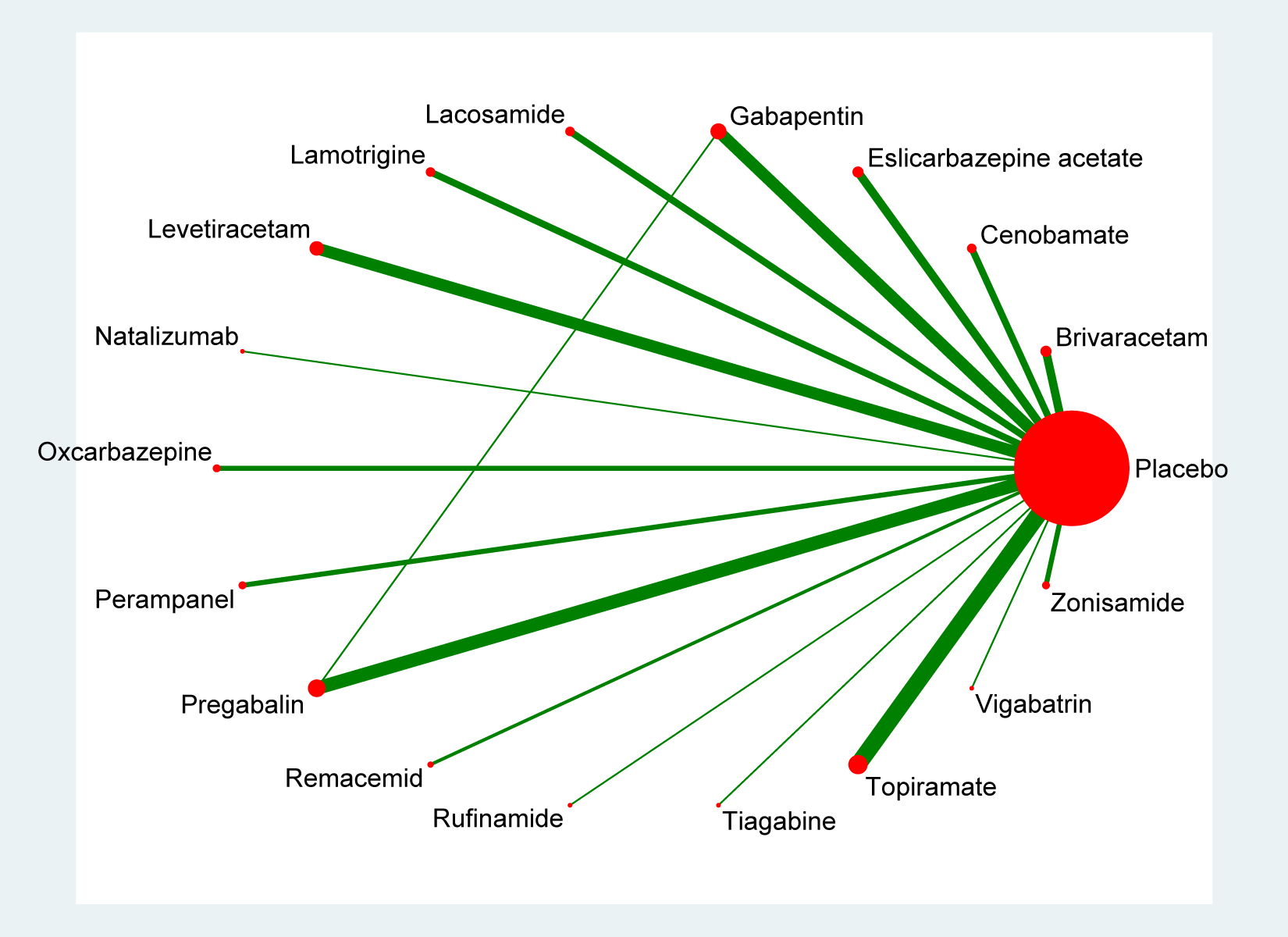
**

**Supplementary Figure 4** Network plot for ataxia

**
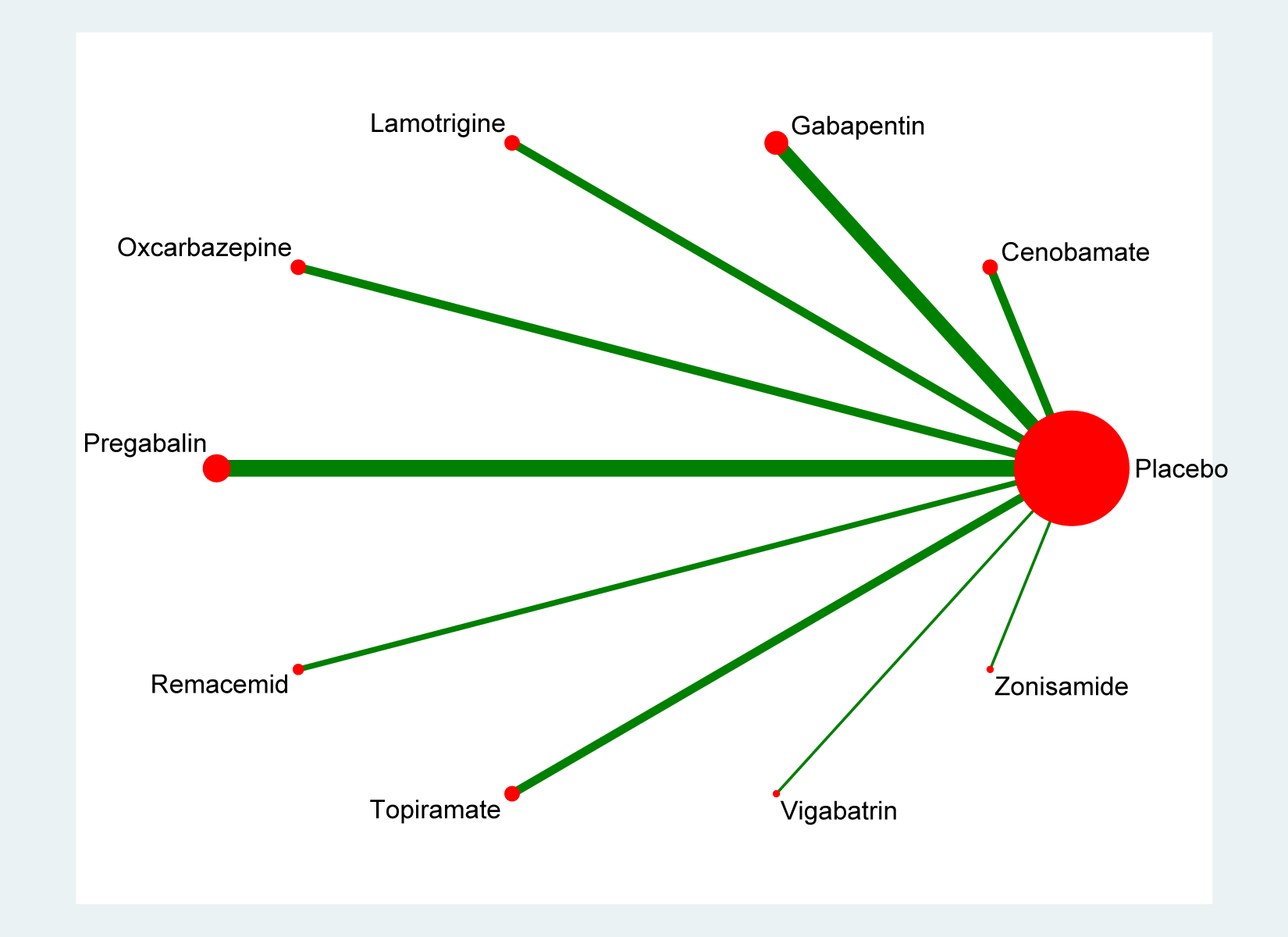
**

**Supplementary Figure 5** Network plot for diplopia

**
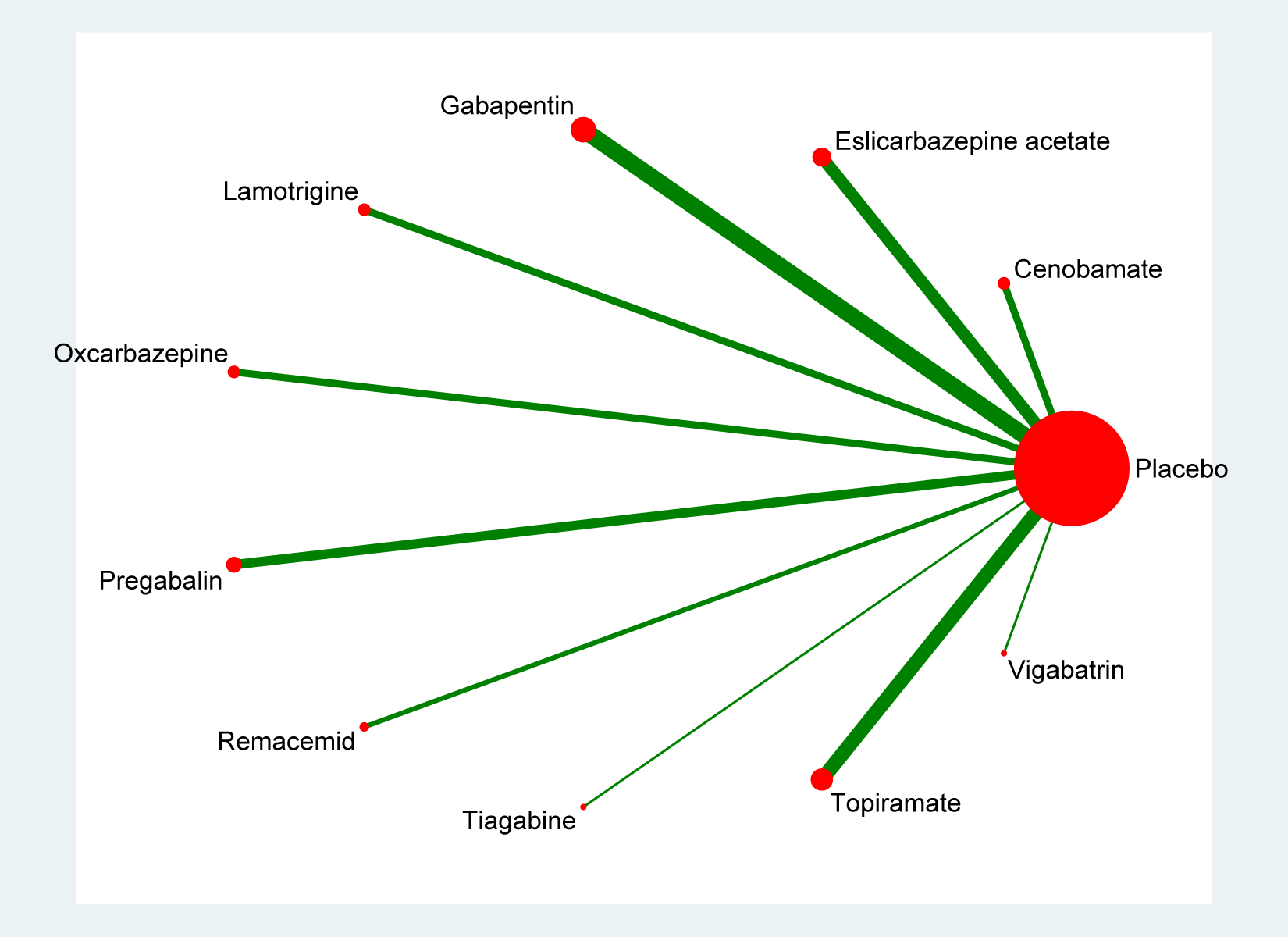
**

**Supplementary Figure 6** Network plot for fatigue

**
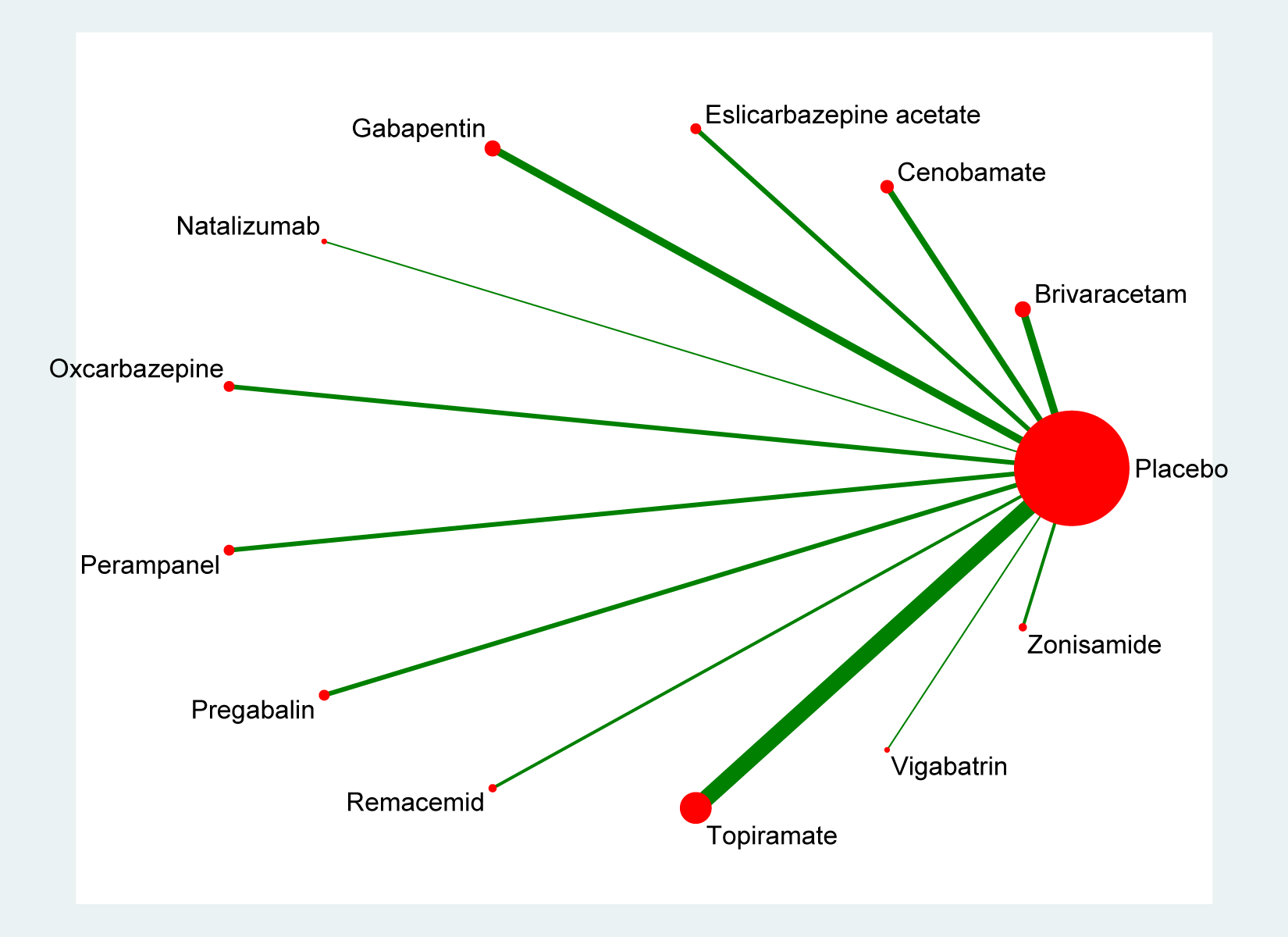
**

**Supplementary Figure 7** Network plot for nausea

**
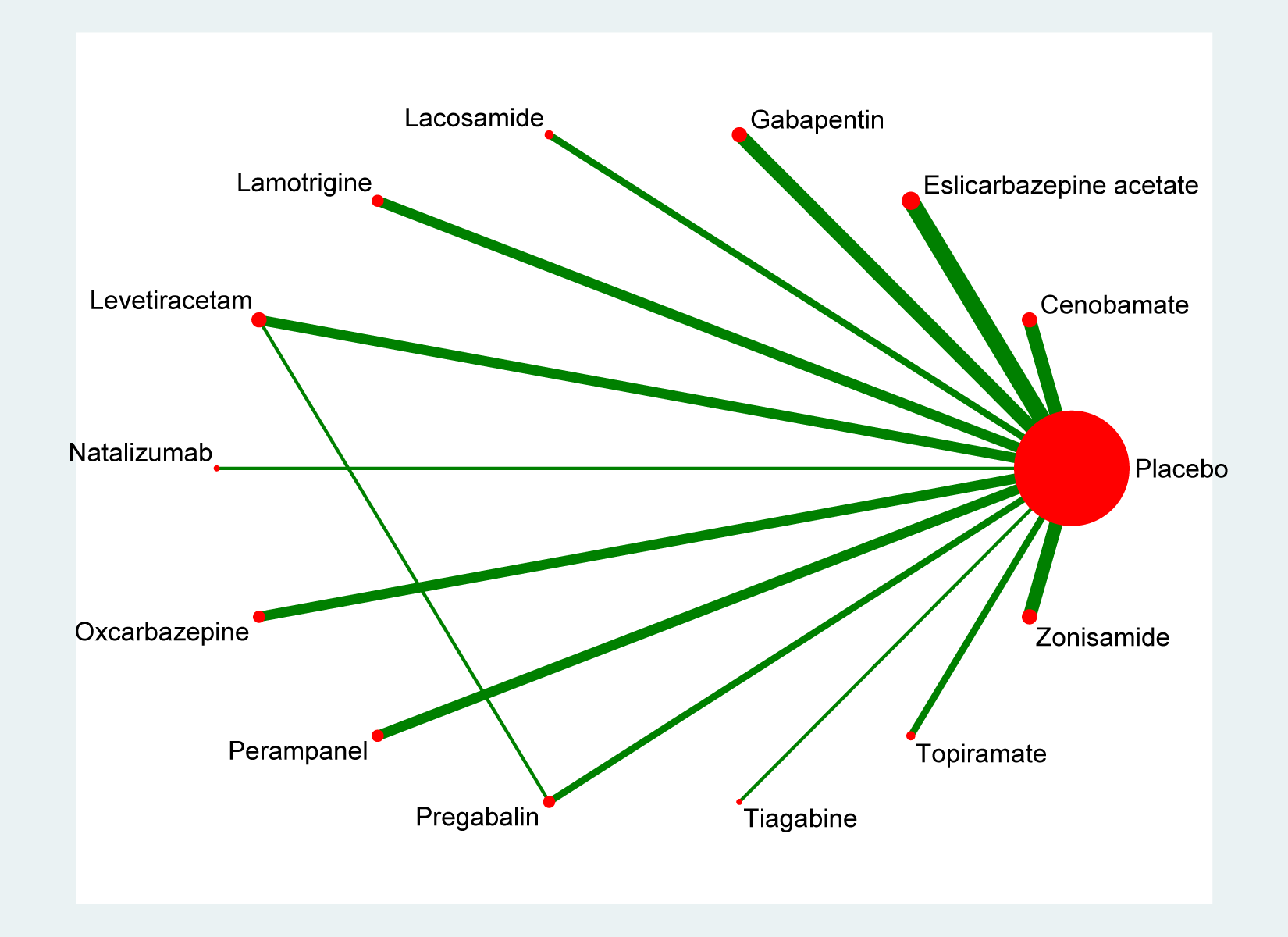
**

**Supplementary Figure 8** Funnel plot for 50% response rate

**
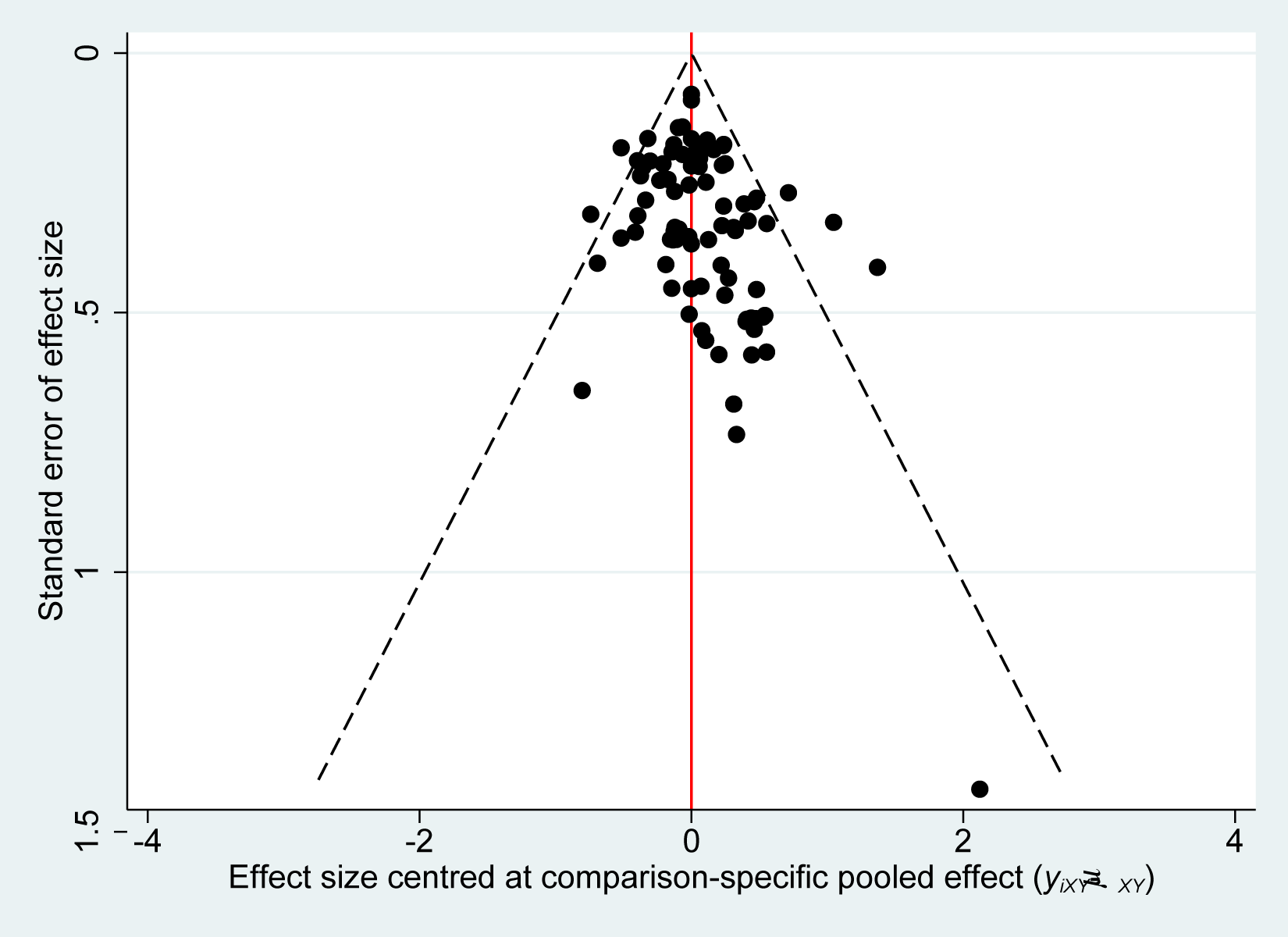
**

**Supplementary Figure 9** Funnel plot for dizziness

**
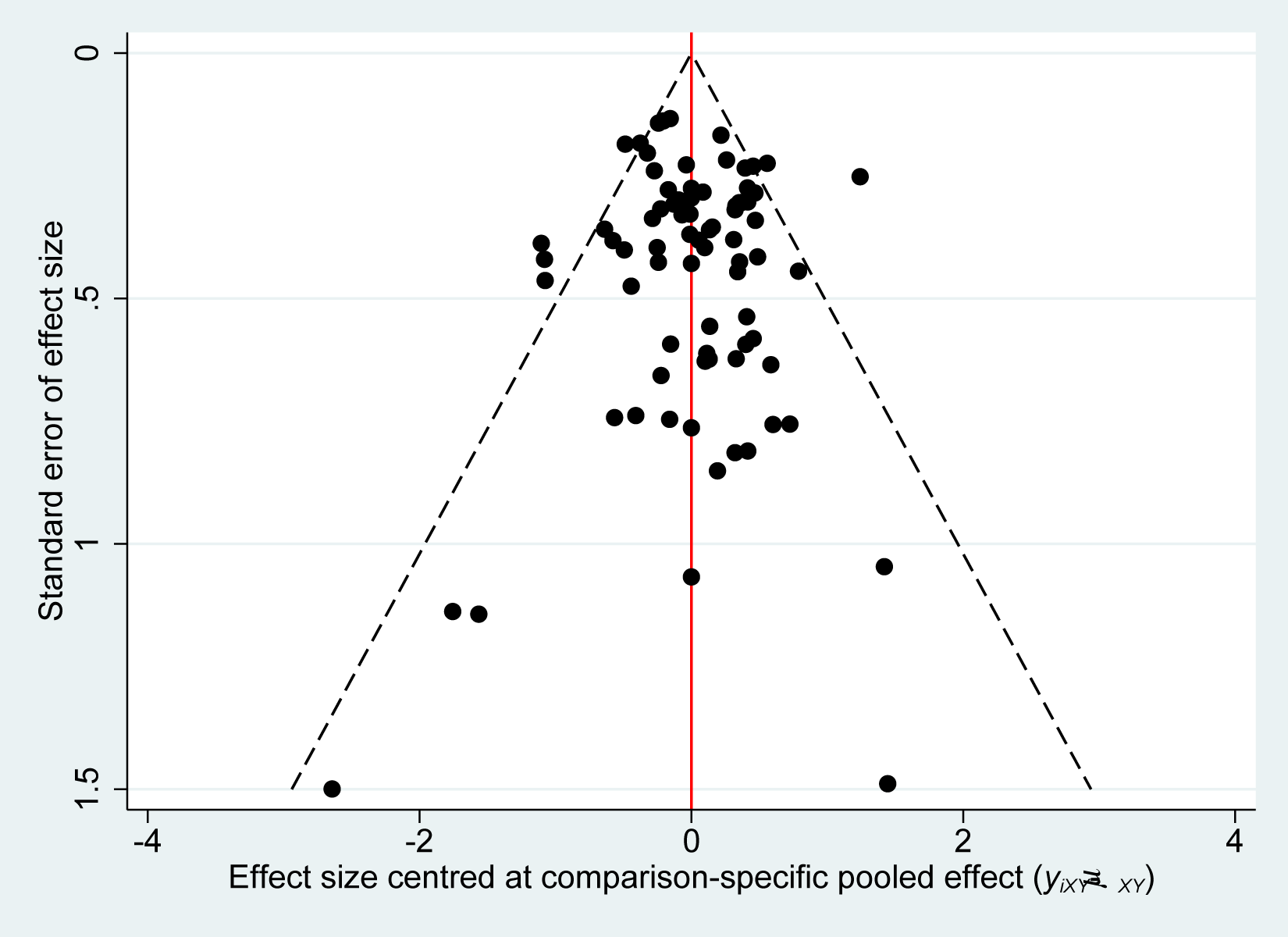
**

**Supplementary Figure 10** Funnel plot for somnolence

**
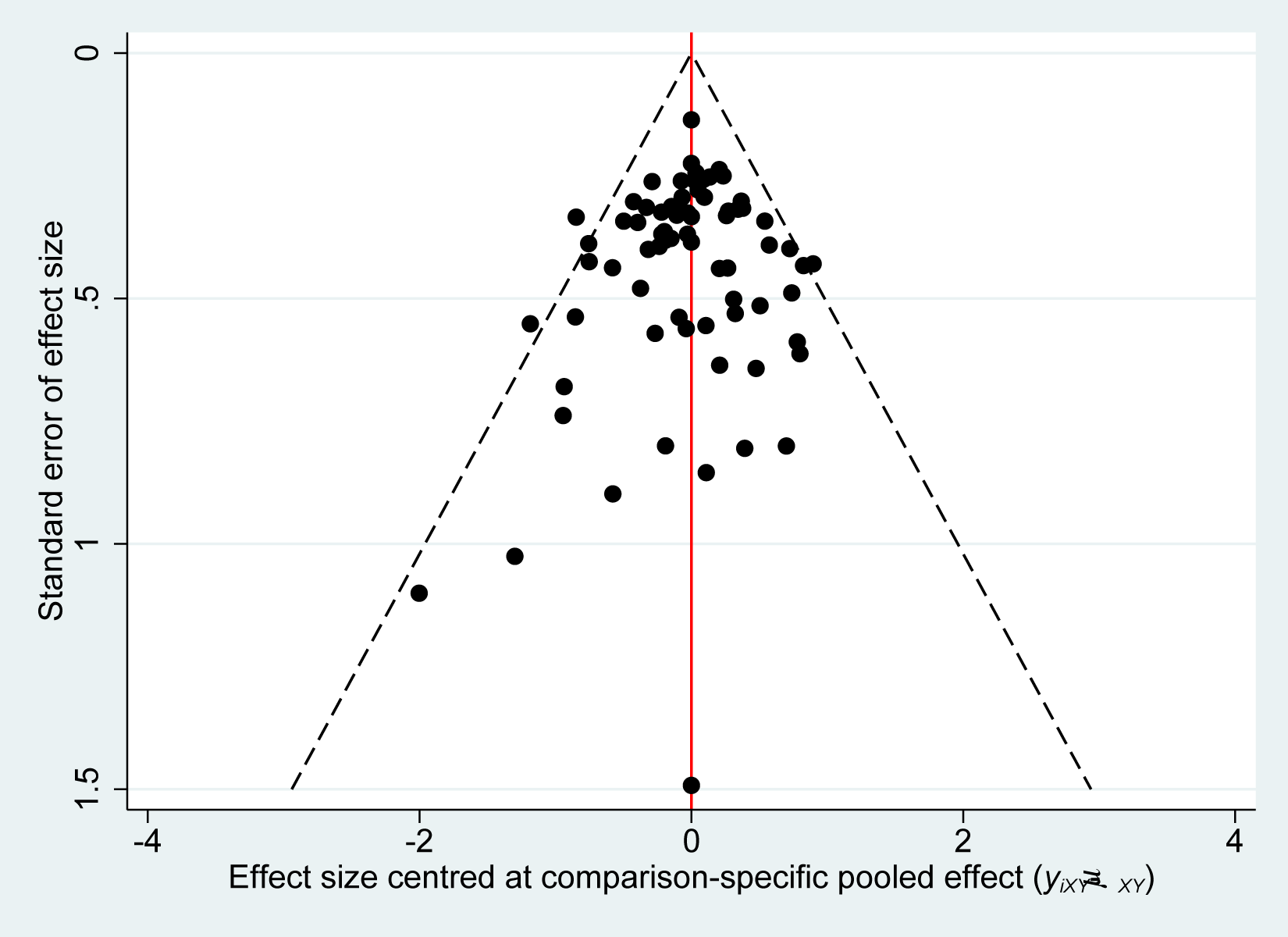
**

**Supplementary Figure 11** Funnel plot for headache

**
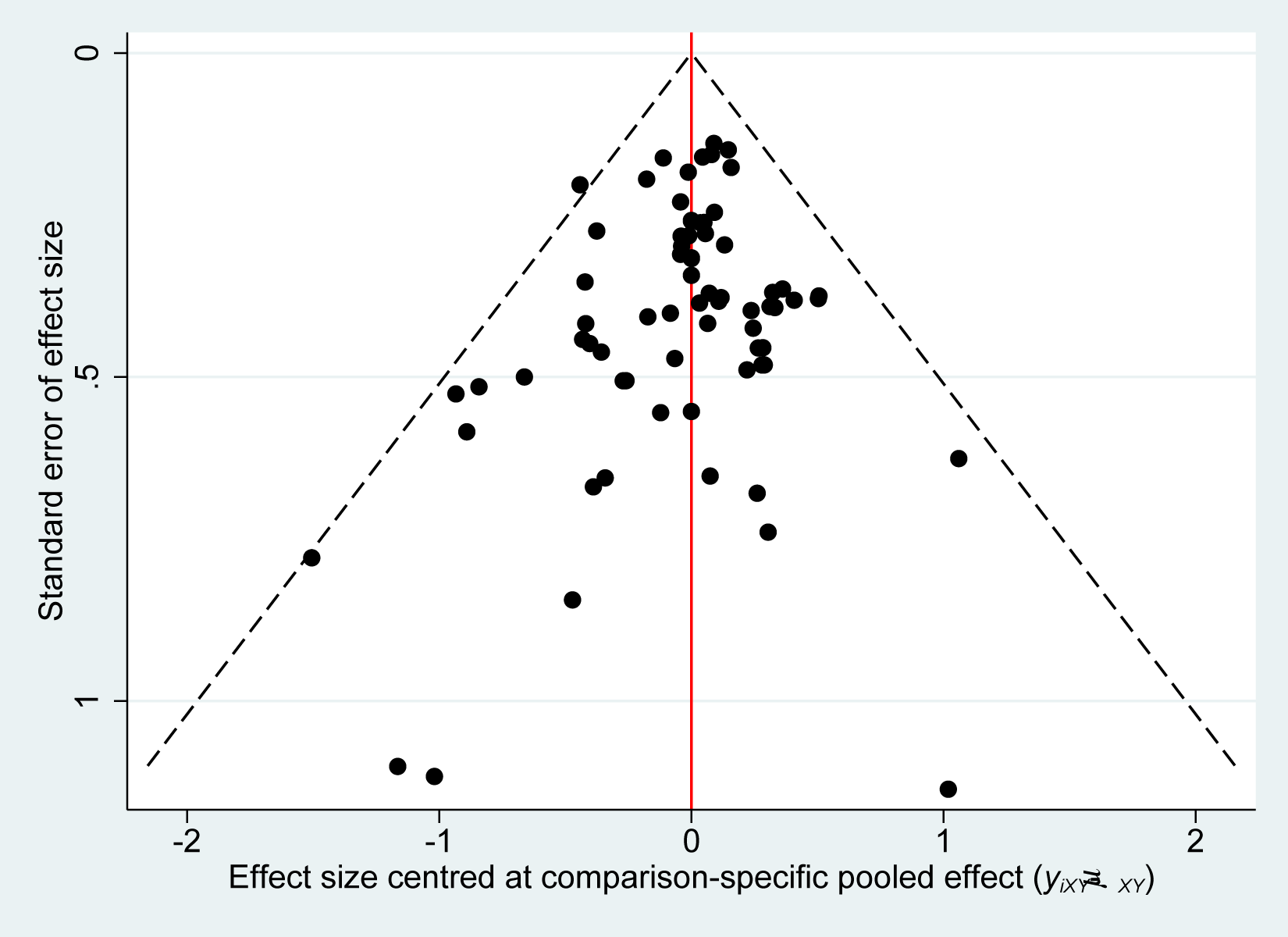
**

**Supplementary Figure 12** Funnel plot for ataxia

**
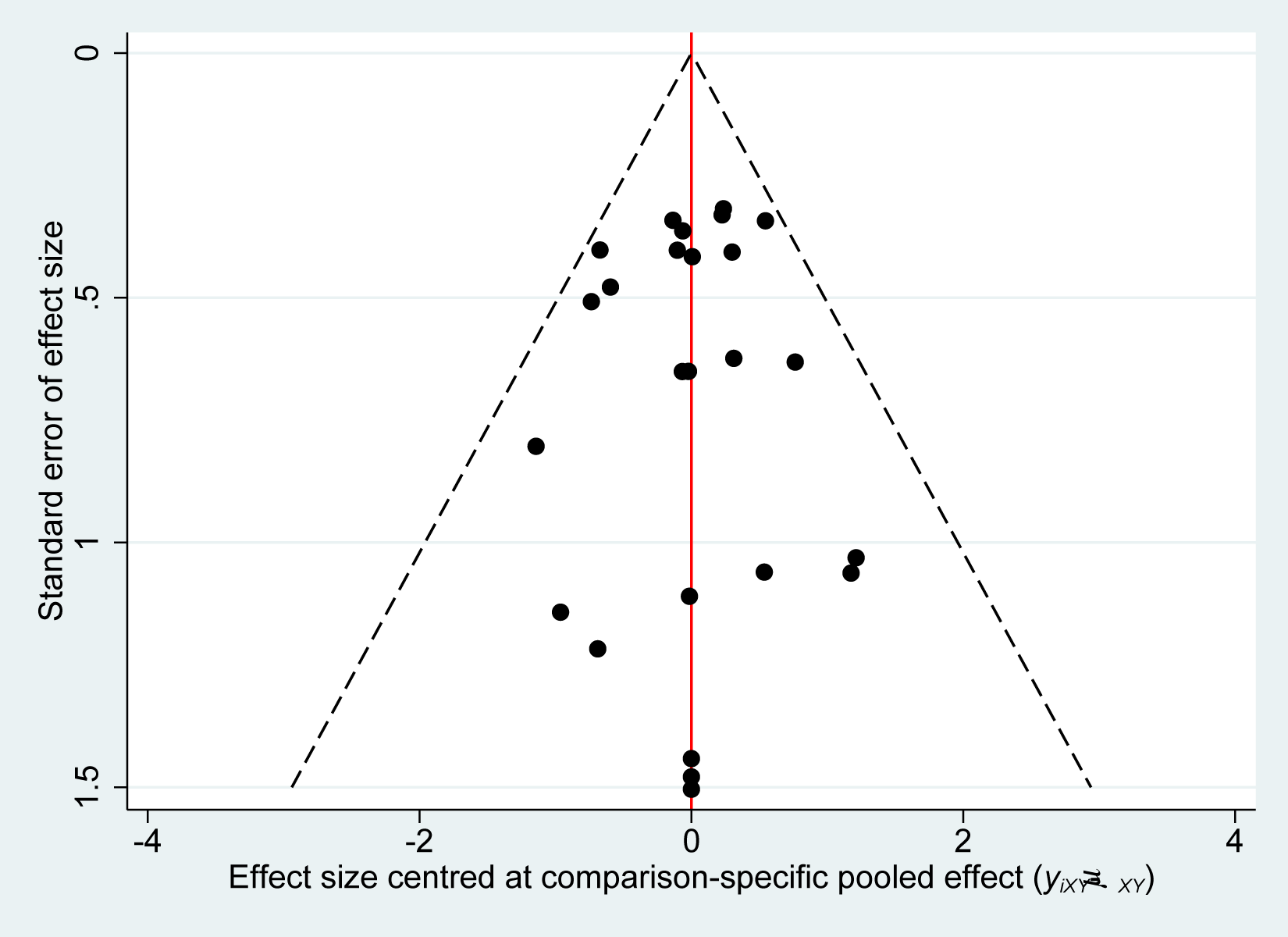
**

**Supplementary Figure 13** Funnel plot for diplopia

**
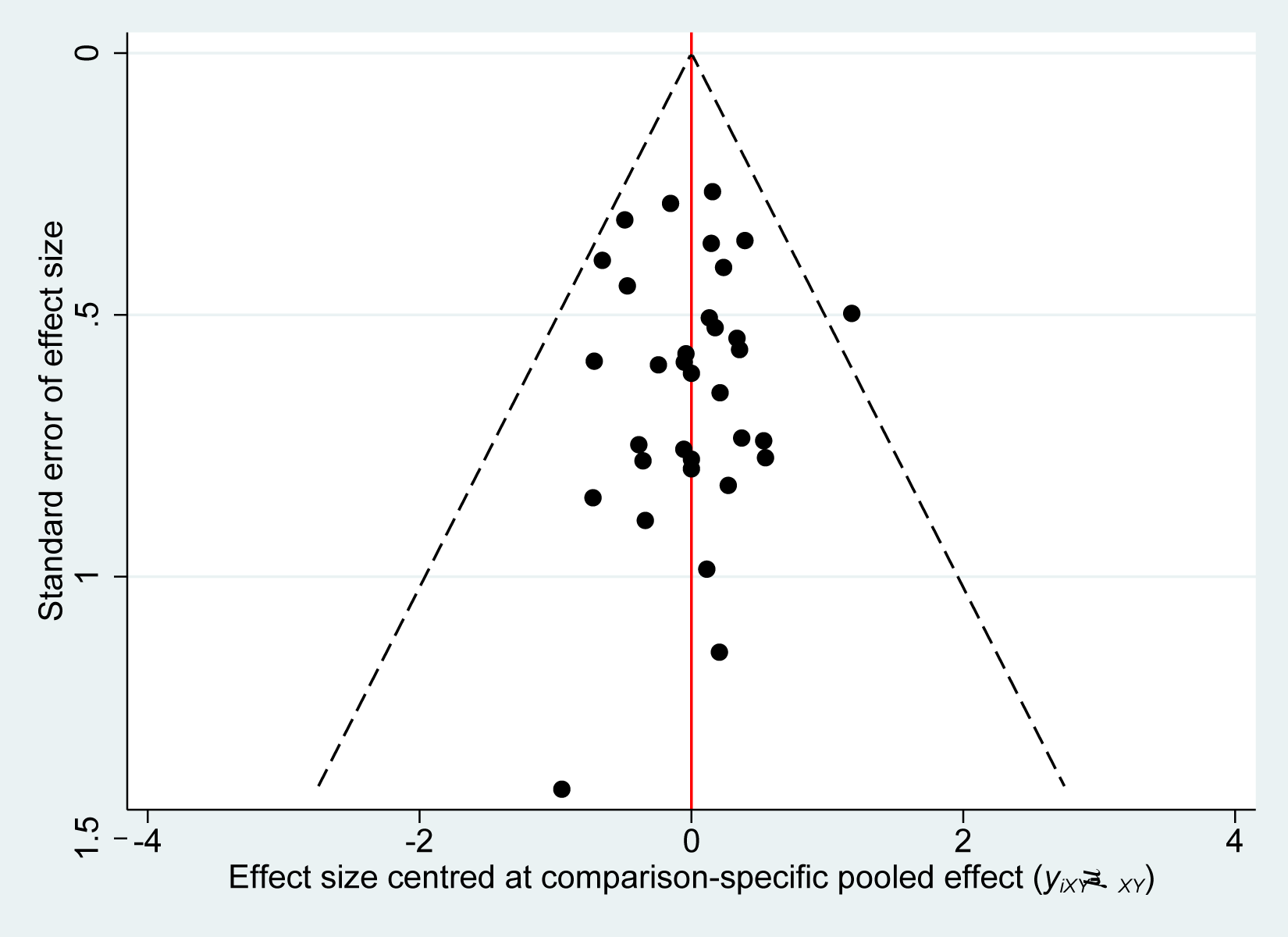
**

**Supplementary Figure 14** Funnel plot for fatigue

**
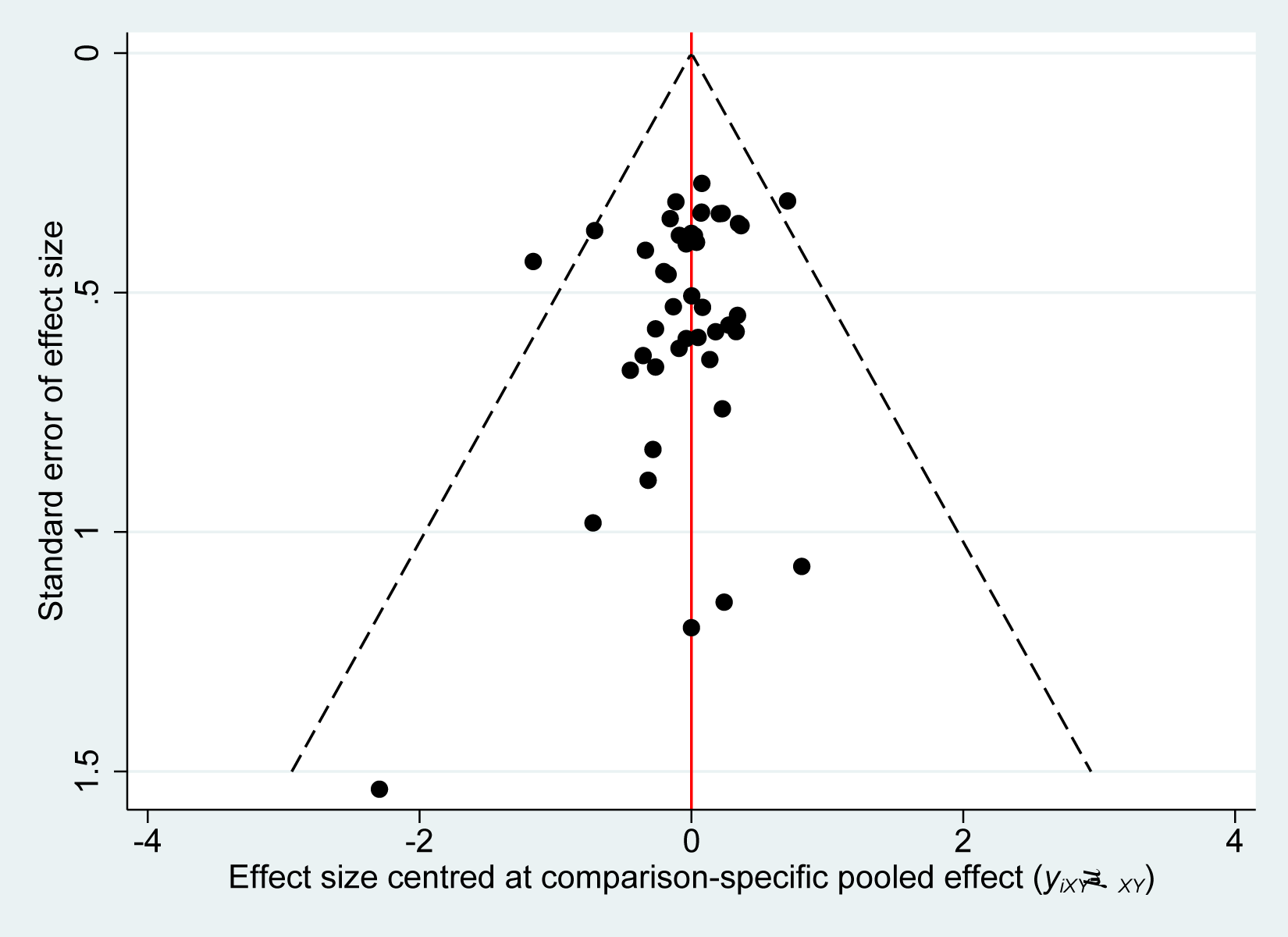
**

**Supplementary Figure 15** Funnel plot for nausea


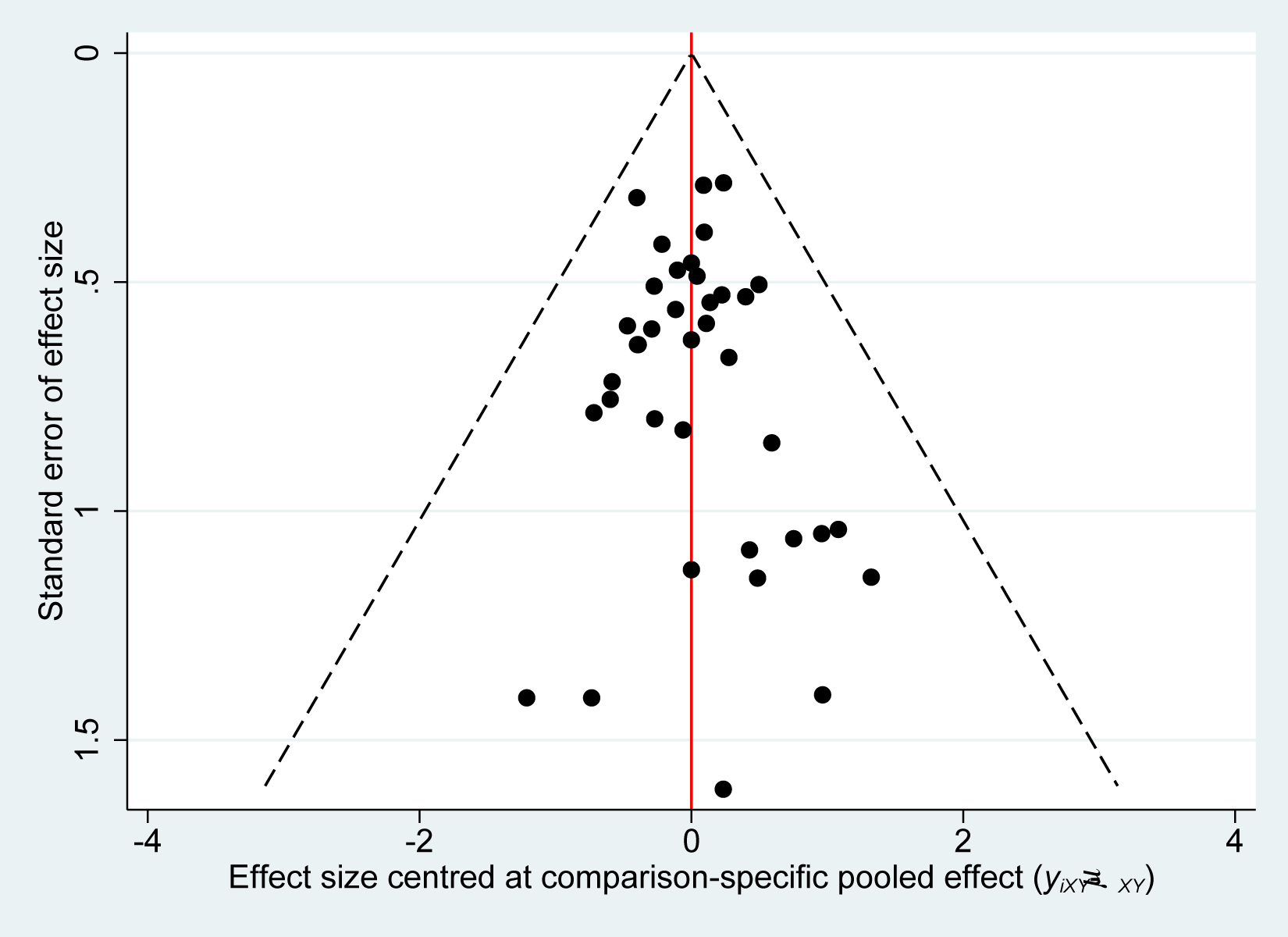

Supplement: Supplementary file 1 [file Supplementaryfile1.docx]
